# Re-evaluating molecular clock maximum age calibrations revives pre-Ediacaran divergence estimates for animals

**DOI:** 10.1126/sciadv.aeg6289

**Published:** 2026-10-02

**Authors:** Orin M. Lole Durbin, Eliel S.C. Anttila, Derek E.G. Briggs, Francis A. Macdonald, Ross P. Anderson

**Affiliations:** ^1^ Department of Earth Sciences, University of Oxford, Oxford OX1 3AN, UK.; ^2^ Department of Geosciences, Virginia Tech, Blacksburg, Virginia 24061, USA.; ^3^ Department of Earth and Planetary Sciences, ETH Zürich, Zürich 8092, Switzerland.; ^4^ Department of Earth and Planetary Sciences and Yale Peabody Museum, Yale University, New Haven, CT 06511, USA.; ^5^ Department of Earth and Planetary Sciences, University of California–Berkeley, Berkeley, CA 94720, USA.; ^6^ Museum of Natural History, University of Oxford, Oxford OX1 3PW, UK.

## Abstract

Divergent models persist for the emergence of animals. Lipid biomarkers suggest animals originated before 635 million years ago (Ma), but their body fossil record extends to only 574 Ma. Molecular clocks might differentiate between these records, but we demonstrate how their accuracy and precision hinge on maximum age calibrations. We evaluate the Ediacaran Weng’an Biota (China) as a maximum calibration utilizing microfossils from the Kheseen Biota (Mongolia). These two biotas have similar preservation and microfossil assemblages, containing no definitive animals. The Kheseen Biota, deposited at a time (∼550–526 Ma) when animals were undoubtedly present, questions the validity of the Weng’an calibration. We discuss the uncertainty in maximum calibration strategies and propose multiple pre-Ediacaran deposits with favorable taphonomy, preserving both non-animal body fossils and biomarkers, as currently the most reliable calibrations. Our analyses support a late Tonian to mid-Cryogenian (∼800–700 Ma) origin for Metazoa.

## INTRODUCTION

The timing of the origin of animals on Earth has puzzled scientists for centuries. Unambiguous macrofossil evidence of animals extends only to the earliest examples of the Ediacara Macrobiota, dated to ∼574 million years ago (Ma) (*1*), and the microfossil record likewise does not record definitive animals until the Ediacaran (*2*). Lipid biomarkers, however, suggest animals were present during the terminal 10 to 20 million years (Myr) of the Cryogenian (717 to 635 Ma) (*3*); recent analyses indicate that signatures specific to sponges (Porifera) are preserved in sufficient abundance in Neoproterozoic rocks to support their presence (*4*). This represents a stark dichotomy. Lipid biomarkers indicate that crown Metazoa evolved at least 75 Myr before their first appearance as fossils (*5*).

Molecular clocks have been used to test these competing hypotheses (*6*–*9*), but they rely on the integration of paleontological and geological data to estimate lineage divergence times (*10*). Minimum and/or maximum age calibrations, based on evidence from the rock record, must be assigned to individual nodes. Unambiguous animal body fossils within the Ediacara Macrobiota provide a hard minimum calibration of 574 Ma for crown Metazoa (*11*). Animals must have evolved before this date. Determining a maximum calibration for animals (a date at which they had not yet evolved) is a more complex task (*12*). Maximum calibrations are based on evidence-of-absence (*13*), i.e., a geological unit that combines the ecologic, biogeographic, geologic, and taphonomic conditions for animal fossils (body, trace, or biomarker) but yields no fossil representatives. A variety of late Tonian (∼850 to 717 Ma) assemblages (e.g., Bitter Springs Group, Australia; Svanbergfjellet Formation, Svalbard) have previously been used as maximum calibrations on crown Metazoa [e.g., (*14*, *15*)]. These microfossil assemblages contain no recognizable animal body fossils despite being preserved under taphonomic conditions that promote the survival of soft and fragile animal tissues [e.g., carbonaceous compressions associated with early diagenetic clays, i.e., Burgess Shale-type (BST) preservation or early silicification (*16*, *17*)].

Using younger maximum calibrations, such as the Lantian and Weng’an biotas of China, brings molecular clock estimates for the antiquity of animals into the early Ediacaran (*11*, *18*) closer to their unambiguous fossil record. The ∼602 ± 7 Ma Lantian Biota (*19*) preserves soft-bodied algae as carbonaceous compressions (similar to Cambrian BST preservation without demonstrated clay associations) but no definitive animal body fossils (*20*), although see (*21*). This deposit has been proposed as a maximum calibration for crown Metazoa based on the assumption that the taphonomic conditions would preserve early animal tissues if these organisms were present (*11*, *18*). Similarly, the phosphatized Weng’an Biota has been proposed as a maximum calibration of 590.4 ± 2.6 Ma (*1*) for crown Eumetazoa and crown Bilateria (*11*) and speculated as a possible maximum calibration on crown Metazoa (*22*). The Weng’an Biota has been studied extensively, but no definitive metazoan, eumetazoan, or bilaterian body fossils have been identified (*22*), although affinities of Weng’an taxa remain controversial (*23*–*25*). Phosphatized Cambrian assemblages in Australia, Mongolia, Siberia, South China, and the United States, however, include unequivocal eumetazoan embryos (*26*–*28*). Hence, the arguable absence of eumetazoans in the Weng’an Biota may indicate that the clade had yet to evolve by the early Ediacaran (*11*).

Assessing the reliability of absence-based evidence, such as the Lantian and Weng’an biotas, might be achieved by comparison with analogous younger assemblages. However, a lack of suitable latest Ediacaran–earliest Cambrian fossil-bearing successions has thus far prevented such an approach for the Weng’an Biota (*29*). The phosphatized Kheseen (previously Khesen) Biota of Mongolia provides an opportunity to test the reliability of the Weng’an calibration. The Kheseen Biota is preserved in phosphorites of the Kheseen Formation (*30*), a ∼150 to 500-m-thick carbonate-dominated succession (Fig. 1). A prominent erosional unconformity separates the base of the Kheseen Formation from the underlying carbonate-rich Khirvesteg Formation (Fig. 1B) that was formed in the aftermath of the Marinoan (∼639 to 635 Ma) Snowball Earth Glaciation. At Kheseen Gol (Fig. 1A), the Kheseen Formation is capped by coarse-grained, fining-upward grainstone beds and a siliciclastic-rich conglomerate, which has yielded a detrital zircon maximum depositional age of 525.19 ± 1.3 Ma (Fig. 1B). The best-preserved microfossils occur in allodapic lithofacies toward the base of the Kheseen Formation (Fig. 1B), where individual fossils occur as allochems in packstone and grainstone beds (*31*, *32*).

**Fig. 1.**
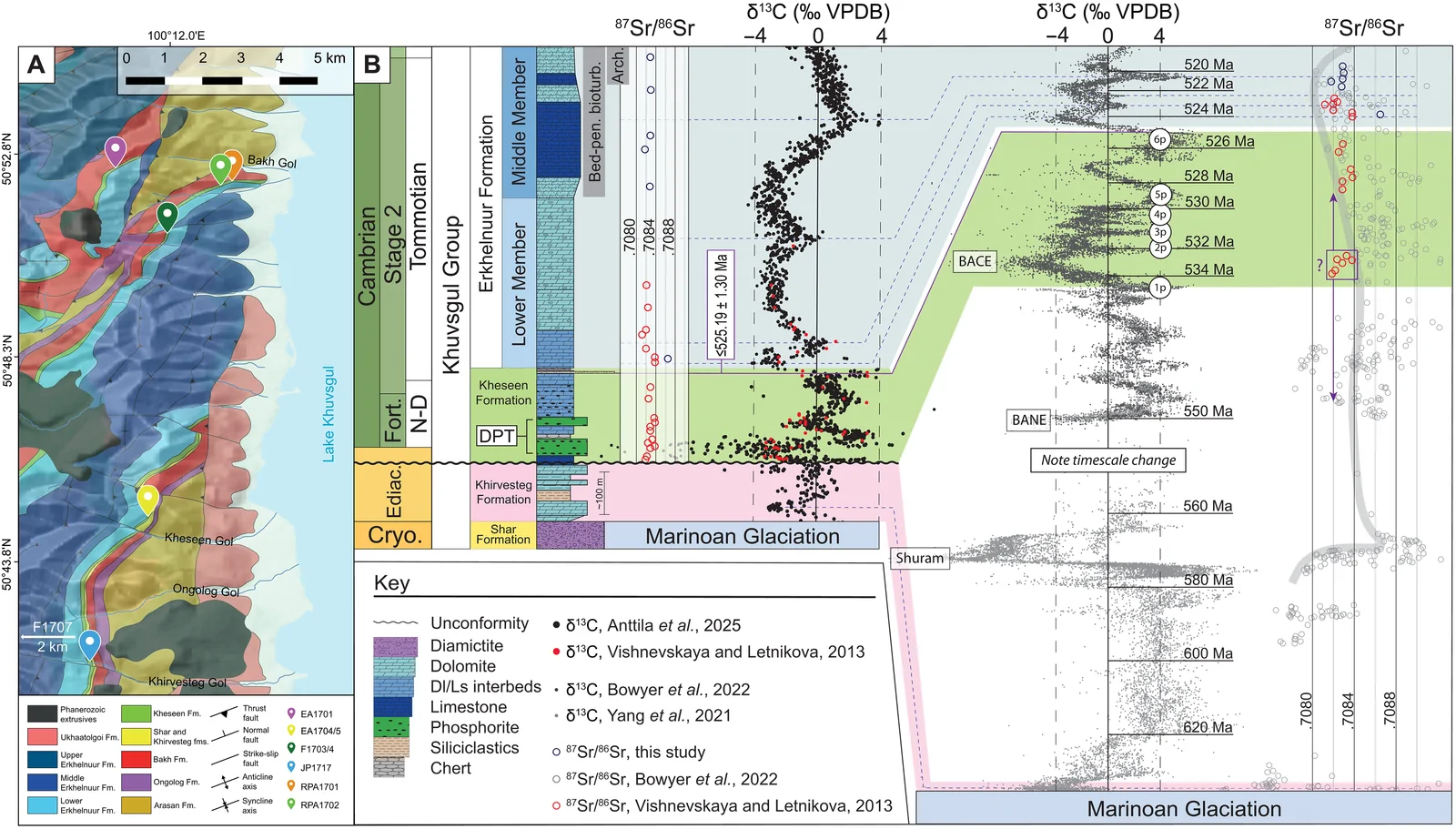
Geological setting of the Kheseen Formation. (**A**) Geological map of the western shore of Lake Khuvsgul, modified from Anttila *et al.* (*35*). Sample localities are labeled. (**B**) Generalized lithostratigraphy and chemostratigraphy of the Kheseen Formation and adjacent strata of the Khuvsgul Group. Correlations between ^87^Sr/^86^Sr and δ^13^C chemostratigraphy of the Khuvsgul Group (at center left) and global compilations (at right) are depicted as a fence diagram. Evidence for sedimentary condensation, hiatus, and localized remineralization of organic carbon complicates the equivocal correlation of the basal Kheseen δ^13^C nadir with a specific excursion in the Fortunian global δ^13^C compilation. Note that ^87^Sr/^86^Sr data could support either a younger or older correlation (arrows) than the δ^13^C-based age model of (*35*). Together, δ^13^C and ^87^Sr/^86^Sr data suggest that the fossiliferous interval of the Kheseen Formation was deposited post-550 Ma but before 526 Ma, in the latest Ediacaran or early Cambrian. Global compiled chemostratigraphy and associated Ediacaran age model are derived from (*33*); note that we use the Cambrian age model of (*106*). Dl/Ls, dolomite/limestone; Bed-pen. bioturb., bed-penetrating bioturbation; Arch., archaeocyaths; DPT, Doushantuo-Pertatataka-type microfossil assemblage; BACE, BAsal Cambrian carbon isotope Excursion; BANE, BAsal Nama carbon isotope Excursion.

Here, we report Kheseen Biota microfossils and situate them within a refined age model. We use this microfossil assemblage, alongside complimentary evidence from other Neoproterozoic deposits, to assess the reliability of the Weng’an maximum calibration, prompting an exploration of the impact of different maximum calibrations on animal molecular clocks and evaluation of the criteria necessary to choose the most reliable maximum calibrations in future studies.

## RESULTS

### Kheseen Biota age model

Carbonate δ^13^C isotope values through the Kheseen Formation show several peaks and nadirs that can be correlated with global δ^13^C compilations [e.g., (*33*)]. A decrease in δ^13^C from ∼+4 to −5‰ at the base of the Kheseen Formation (Fig. 1B) has previously been proposed as equivalent to either the Ediacaran Shuram (EN3) carbon isotope excursion (*34*) or the BAsal Cambrian carbon isotope Excursion (BACE) (*35*). However, these correlations are equivocal, particularly because the interval with the most negative values hosts evidence of local remineralization of organic matter (*35*). New and existing (*36*) ^87^Sr/^86^Sr data, with values of 0.7083 to 0.7084, support an early Cambrian age of the Kheseen Formation (Fig. 1B), as late Ediacaran seawater ^87^Sr/^86^Sr data are characterized by values above 0.7084 and the lowest (least altered) early Cambrian data are below this threshold (*33*). Alternatively, a correlation between the Kheseen phosphorites and the late Ediacaran Khatyspyt Formation, Siberia (*33*), could be justified on the basis of their similar ^87^Sr/^86^Sr values. The Khatyspyt Formation has been constrained to between ∼550 and 543.9 ± 0.24 Ma (*37*). In this scenario, negative δ^13^C values in the basal Kheseen Formation would be equivalent to post-BANE (BAsal Nama carbon isotope Excursion), pre-BACE (*33*) negative δ^13^C excursions (Fig. 1B). This correlation provides a maximum depositional age of 550 Ma for the Kheseen Formation phosphorites. A proposed older equivalence with the Shuram (*34*) derived from a structurally repeated section is precluded by the absence in the Kheseen Formation of the positive trend in ^87^Sr/^86^Sr to values greater than 0.7088 (Fig. 1B) that is observed elsewhere alongside the strong negative excursion in δ^13^C.

δ^13^C and ^87^Sr/^86^Sr chemostratigraphic data support previously proposed lithostratigraphic correlations (*38*) between the Kheseen Formation and phosphorite-bearing strata of the Zavkhan Terrane, southwest Mongolia. Concentrated, hardground-bearing phosphorites of the Kheseen Formation are likely condensed equivalents of the more diffuse, lower-grade phosphatic intervals of the basal Zuun-Arts and Bayangol formations. The stratigraphy of the Zavkhan and Khuvsgul terranes reflects a continuous Neoproterozoic to early Cambrian margin (*35*) with a substantial Ediacaran unconformity resulting in the absence of ∼600 to 550 Ma strata (Fig. 1B) (*39*). The Zuun-Arts and Bayangol formations yield similar ^87^Sr/^86^Sr values to the Kheseen Formation (*40*) and provide additional biostratigraphic support (*41*) for a latest Ediacaran to early Cambrian age. The Bayangol Formation hosts a variety of small shelly fossils (SSFs) that occur stratigraphically above a nadir in δ^13^C in the Zuun-Arts Formation, which has been interpreted as correlative to the BACE (*41*).

The syn-BACE interpretation of the Zuun-Arts δ^13^C nadir and the lithostratigraphic similarities between the basal Kheseen and Zuun-Arts formations provide supporting evidence for the broad correlation of the lower Kheseen Formation δ^13^C excursions with those in the Fortunian Stage. Multiple hardgrounds within this stratigraphic interval suggest that time could be significantly condensed in the Kheseen Formation phosphorites. Thus, correlating to specific δ^13^C negative excursions between 534 and 526 Ma is challenging (Fig. 1B), but taking the constraints described above, we suggest that the strata hosting the Kheseen Biota were likely deposited between 534 and 526 Ma and must be younger than 550 Ma (Fig. 1B).

### New fossil taxa from the Kheseen Biota

New fossil findings from previously unexplored stratigraphic sections along the western shore of Lake Khuvsgul (Fig. 1A) increase the known diversity of phosphatized microfossil taxa from the Kheseen Biota to 16 acritarch species, 8 newly identified here. Previous studies (*31*, *32*) recovered the acanthomorphs *Appendisphaera grandis*, *Appendisphaera fragilis*, *Appendisphaera tenuis*, *Cavaspina basiconica*, *Variomargosphaeridium aculeiparvum*, and *Variomargosphaeridium gracile*. We add *Appendisphaera* sp., *Asterocapsoides wenganensis*, *Knollisphaeridium maximum*, *Mengeosphaera reticulata*, *Sinosphaera* sp., and *Variomargosphaeridium* sp. The diversity of *Megasphaera* known from the Kheseen Biota, previously confined to *Megasphaera minuscula* and *Megasphaera puncticulosa* (*32*), is also expanded to include *Megasphaera inornata* and *Megasphaera ornata*, in addition to a previously undocumented form*.* A report from an exposure of the Kheseen Formation ∼150 km to the south of Lake Khuvsgul describes 12 acritarch species (*34*), including *A. grandis*, *A. tenuis*, *A. wenganensis*, and *K. maximum* recognized here from the north. None of the taxa in this work or in previous studies (see the Supplementary Materials for additional discussion), either at Lake Khuvsgul or the locality to its south, can be confidently allied with Metazoa (*5*, *11*).

A single specimen of *Appendisphaera* sp. (Fig. 2A) is represented by a fragment of vesicle wall attached to a detrital phosphate grain. It is identified as a likely new species of *Appendisphaera* based on the presence of five well-preserved, long (∼100 μm), thick (3 to 6 μm), and tapering processes. These processes are longer than typical (commonly up to 50 μm) for this genus, supporting the establishment of a new species. Although the Kheseen specimen aligns with the diagnosis of *Appendisphaera*, its fragmentary nature and preservation via phosphatization make comparisons challenging and the designation of a new species tentative.

**Fig. 2.**
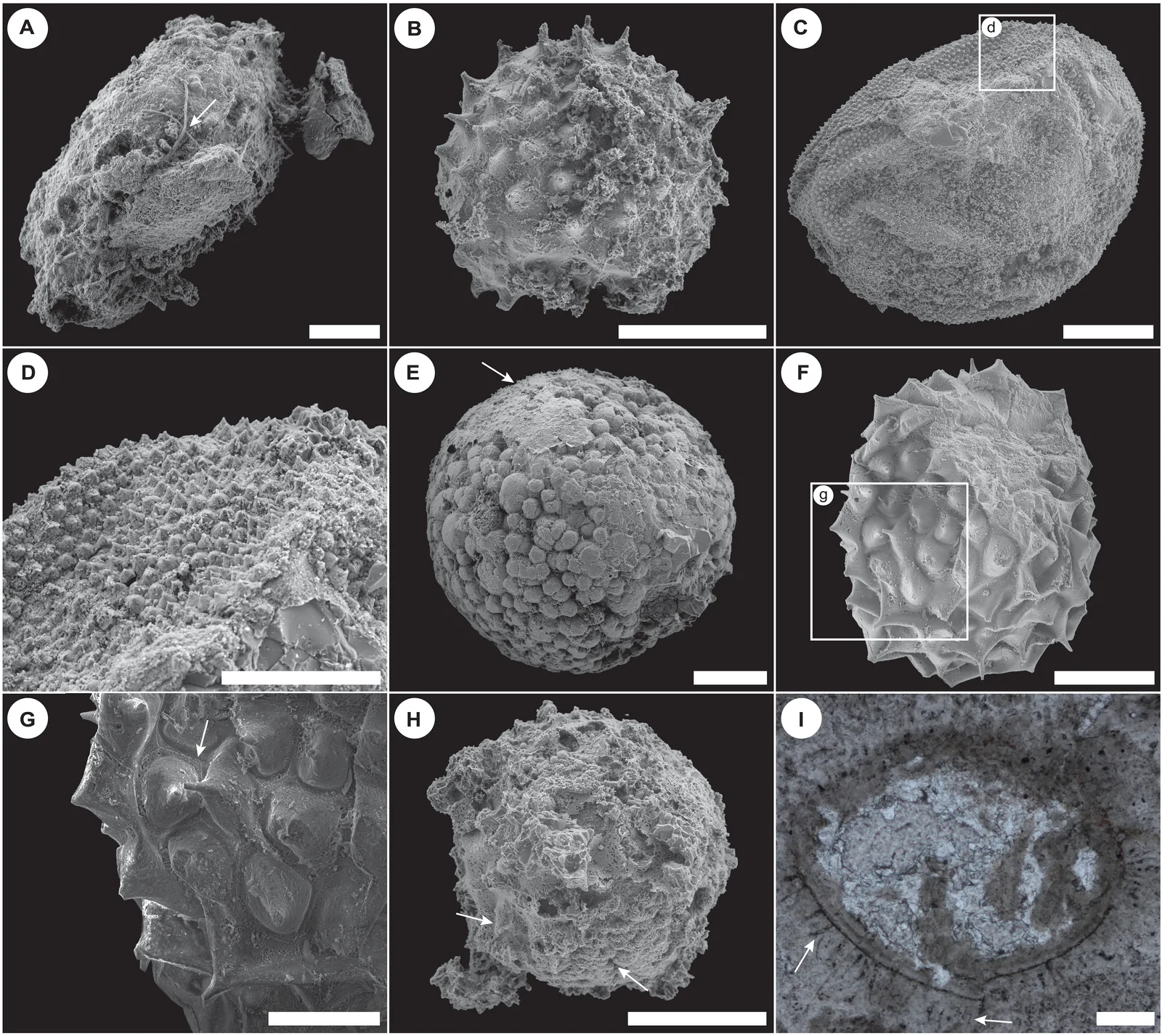
Acanthomorphic acritarchs of the Kheseen Biota. (**A**) Tentatively identified new species of *Appendisphaera* with long tapering processes (arrow), Yale Peabody Museum (YPM) specimen IP 258106. (**B**) *Asterocapsoides wenganensis*, YPM IP 258043. (**C** to **E**) *Knollisphaeridium maximum*. (C) YPM IP 258165. A magnified view of the processes is shown in (D). (E) YPM IP 258055, primarily exhibiting internal morphology with only a portion of the external vesicle preserved (arrow). (**F**) *Mengeosphaera reticulata*, YPM IP 258034. A magnified view illustrating the polygonal fields surrounding the processes (arrow) is shown in (**G**). (**H**) *Sinosphaera* sp., YPM IP 258044, with conical and dome-like processes (arrows). (**I**) *Variomargosphaeridium* sp., YPM IP 258281, with branching processes (arrows), T44/0. England Finder coordinates are given for specimens in thin section. Scale bars, 100 μm (A, B, C, E, F, H, and I) and 50 μm (D and G).

Two specimens are identified as *Asterocapsoides wenganensis* (*42*) based on their broad, slightly deflated conical processes, 18 to 29 μm in length, that ornament vesicles ∼200 μm in diameter (Fig. 2B). *A. wenganensis* can be challenging to differentiate from *Cavaspina*, which also occurs in the Kheseen Formation (*31*, *32*) but differs in its shorter (up to 15 μm) and narrower (∼3-μm basal width) processes (*43*).

*Knollisphaeridium maximum* (*44*) is represented by two specimens (Fig. 2, C to E) identified by their vesicle size (∼375 μm) and densely spaced, acutely conical processes 2 to 5 μm in length. In one case (Fig. 2E), only part of the outer vesicle surface is preserved, exposing an interior morphology of spherical to subspherical structures, 14 to 26 μm in maximum diameter (mean = 21 μm, SD = 3.5 μm, *n* = 45), reminiscent of those present in *Megaclonophycus*-stage *Megasphaera* specimens [figures 7 to 12 in (*45*)].

Five spherical specimens (diameter = 225 to 365 μm) that exhibit biform processes (Fig. 2, F and G) are recognized as *Mengeosphaera reticulata* (*42*). Each process lies within a distinct pentagonal or hexagonal field defined by ridges less than 1 μm wide (Fig. 2G). The expanded basal portion of the processes is obtusely conical and partially inflated with a maximum width of ∼27 to 42 μm. The base extends into an often broken apical spine between 10 and 24 μm in length.

A single specimen, with closely spaced dome-like and conical processes (Fig. 2H), is identified as *Sinosphaera* sp. This genus encompasses small spheroidal vesicles with densely distributed, heteromorphic, dome-like, blunt-tipped processes alongside occasional triangular, conical processes (*42*). The specimen is 195 μm in diameter, ornamented by conical processes ∼16 μm in length and basal diameter, and dome-like processes ∼9 μm in diameter. Processes are separated by a narrow gap or basally joined. The morphology and dimensions of the conical processes (2 complete) ornamenting this specimen suggest that it may represent a new *Sinosphaera* species, as they differ morphologically from those of previously described *Sinosphaera*. Other species exhibit large (>50-μm long) conical or cylindrical processes alongside smaller domed or conical ones (*42*).

A likely new species of *Variomargosphaeridium* is represented by a single specimen in thin section (Fig. 2I); a 537-μm diameter vesicle ornamented by hollow processes that branch ∼40% along their length. Well-preserved processes reveal further branching (Fig. 2I). Process lengths vary from 40 to 60 μm, with basal widths of 5 to 8 μm. The processes are reminiscent of those of *V. gracile* [e.g., (*32*)], but this specimen is significantly larger than any other *Variomargosphaeridium* species (*42*).

The Kheseen Formation previously yielded the sphaeromorphs *Megasphaera minuscula*, also recovered during this study (Fig. 3A) and *M. puncticulosa* (*32*). More than 100 examples of *M. inornata* (Fig. 3B) are recognized here, ranging from 404 to 666 μm in maximum diameter. *M. inornata* comprises smooth, spheroidal vesicles 400 to 1100 μm in diameter that may enclose one or more cells (*42*). Kheseen specimens of *M. inornata* reveal multiple internal cellular bodies in thin section; however, the preservation of internal bodies in acid-extracted phosphatized specimens is rare. A single specimen (Fig. 3C) is assigned to *Megasphaera ornata* based on the size of the vesicle (520 μm in diameter) and its ornamented surface; *M. ornata* is characterized by sculptured vesicles that contain at least one internal body. Domes ornament the surface of the Kheseen specimen, consistent with a cerebral-type ornamentation (*42*). Another *Megasphaera* specimen (Fig. 3D) exhibits an irregular pattern of linear ridges, reminiscent of textures resulting from contraction of *M. inornata* vesicles in the Kheseen Formation and the Weng’an Biota [figure 3 in (*45*)]. In contrast to deformed examples, this Kheseen specimen remains spherical and may instead represent an additional *M. ornata* morphology.

**Fig. 3.**
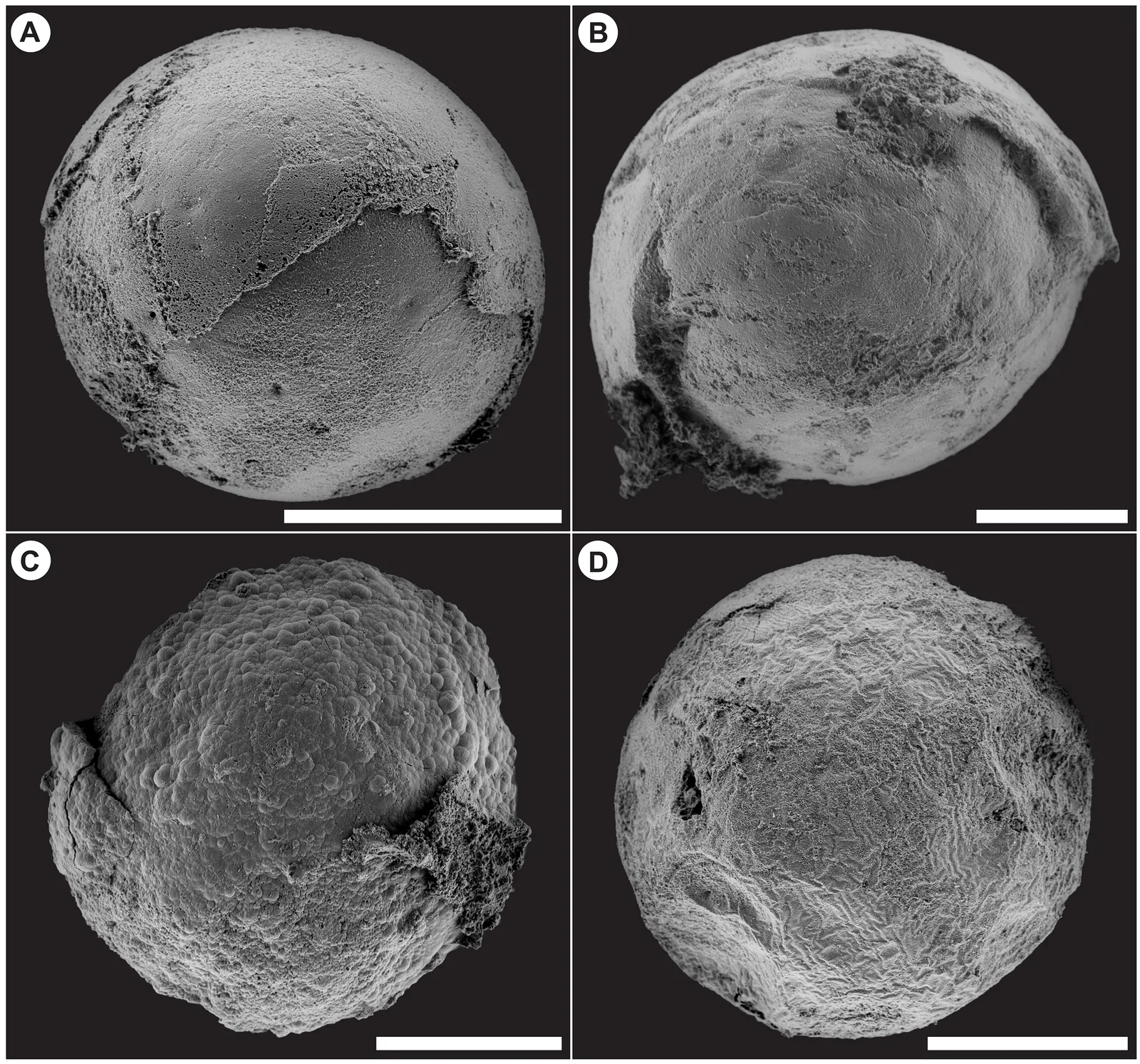
Embryo-like microfossils of the Kheseen Biota. (**A**) *Megasphaera minuscula*, YPM IP 258167. (**B**) *M. inornata*, YPM IP 258201. (**C**) *M. ornata*, YPM IP 258250. (**D**) *Megasphaera* sp., YPM IP 258182, ornamented with irregular ridges. Scale bars, 200 μm.

### Maximum calibration sensitivity analysis

Using the Weng’an Biota as a maximum calibration for crown Metazoa, rather than the Lantian Biota (*11*), resulted in a 590.4 ± 2.6 Ma estimate for the last metazoan common ancestor (Fig. 4A), at least 60 Myr younger than its earliest occurrence as suggested by lipid biomarkers (∼650 Ma) (*46*). This age implies minimal time between the origin of metazoans and the first appearance of eumetazoans and bilaterians in the fossil record. The earliest eumetazoan body fossils [e.g., *Fractofusus andersoni* (*47*)] occur in the ∼574 Ma Ediacaran Avalon assemblage, while the earliest evidence for Bilateria, such as trace fossils made by *Ikaria* and the body fossil *Kimberella*, appears ∼560 to 555 Ma (*48*); *Kimberella* has recently been reported from the Blueflower Formation (*49*), northwest Canada, indicating its range may extend to 564.3 Ma (*50*).

**Fig. 4.**
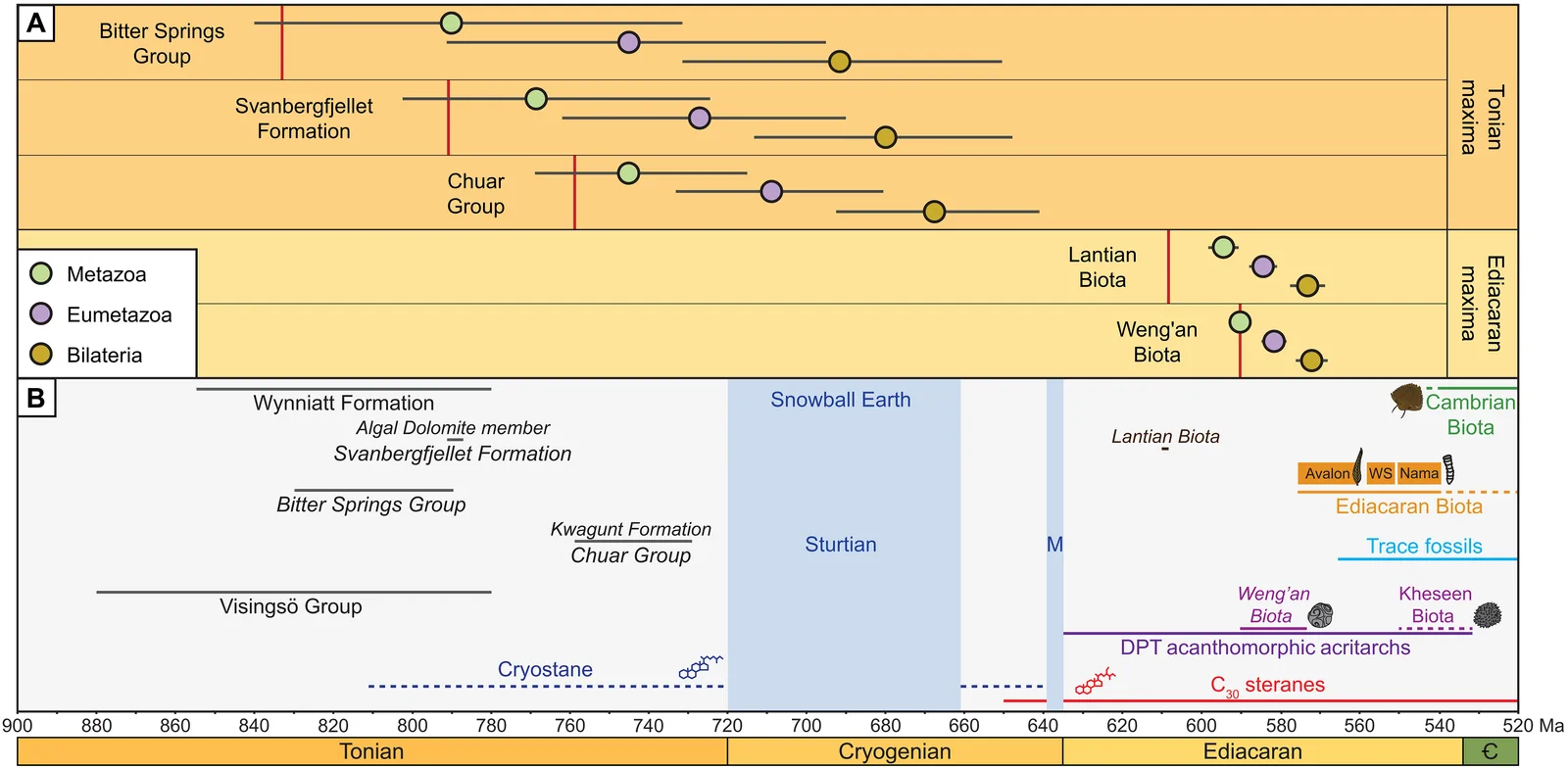
Comparison of recovered animal divergence estimates with varying maximum calibrations. (**A**) From top to bottom, the Tonian Bitter Springs Group, Svanbergfjellet Formation (Algal Dolomite member), and Chuar Group (Kwagunt Formation), and the Ediacaran Lantian (*11*) and Weng’an biotas are used as maximum calibrations. Employed maximum age constraints are indicated by vertical red lines. Divergence estimates for Metazoa (green), Eumetazoa (purple), and Bilateria (tan) are shown as circles with corresponding 95% HPD intervals represented by gray bars. The minimum calibrations of (*11*) were held constant in all analyses. (**B**) Temporal extent of key animal fossil assemblages, groups, and biomarker signatures. The assemblages used as maximum calibrations in (A) are italicized. M, Marinoan Glaciation; DPT, Doushantuo-Pertatataka-type; WS, White Sea.

A Tonian maximum calibration for crown Metazoa provides considerably older estimates for the origin of the clade (Fig. 4A). Tonian deposits (Fig. 4B), such as the Svanbergfjellet Formation (791.1 ± 4.9 Ma) (*51*) or Bitter Springs Group (∼830 Ma) (*15*), have been used as maximum calibrations for animals based on their lack of animal body fossils despite suitable taphonomic conditions for their preservation (*52*). Applying a Svanbergfjellet Formation (Algal Dolomite member) maximum calibration results in the following divergence estimates: Metazoa = 771.7 ± 39.6 Ma; Eumetazoa = 729.7 ± 36.5 Ma; and Bilateria = 681.8 ± 33.3 Ma (Fig. 4A). A Bitter Springs Group maximum calibration produces estimates of 790.6 ± 53.7 Ma, 745.0 ± 48.4 Ma, and 692.2 ± 41.3 Ma for Metazoa, Eumetazoa, and Bilateria, respectively (Fig. 4A).

Younger Tonian assemblages could also be used as maximum calibrations (Fig. 4). The Chuar Group, USA, contains no recognizable animals while preserving a diverse assemblage of macroscopic and microscopic body fossils (*53*, *54*) similar in diversity and preservational fidelity to the Svanbergfjellet Formation, albeit without geochemically demonstrated BST preservation (*52*). Within the Chuar Group, the Kwagunt Formation (maximum age of ∼751.0 ± 7.6 Ma) (*55*) also preserves abundant lipid biomarkers (*56*), none of which are definitive animal signatures (*57*). Cryostane (26-methylcholestane) preserved within the Kwagunt Formation has been suggested to be an animal sterane marker (*58*); however, cryostane sterols have not been reported from modern animal taxa, and this signature is currently regarded as insufficient evidence for the presence of Metazoa (*46*). Applying a Kwagunt Formation maximum calibration results in the following divergence estimates: Metazoa = 745.8 ± 27.0 Ma, Eumetazoa = 709.0 ± 26.4 Ma, and Bilateria = 667.4 ± 25.8 Ma (Fig. 4A).

Additional analyses, designed to test the impact of pre-Tonian constraints on animal evolution, were conducted using the Lakhanda Group, Siberia [another microfossil assemblage with no recognizable animals (*59*) but suitable, i.e., BST preservation, taphonomic conditions (*52*)] and the Great Oxygenation Event (GOE) [before which environmental conditions may have precluded animal physiology (*60*)], dated to ∼1000 (*61*) and ∼2300 Ma (*62*), respectively, as maximum calibrations. These analyses produced near identical divergence estimates (table S2) of 814 ± 90 Ma for Metazoa, 764 ± 75 Ma for Eumetazoa, and 705 ± 55 Ma for Bilateria.

Our analyses demonstrate that the choice of maximum calibration plays an important role in modeling divergence estimates (Fig. 4). When the crown Metazoa maximum calibration was varied by 240 Myr (Ediacaran versus Tonian), the divergence estimates for Metazoa, Eumetazoa, and Bilateria correspondingly varied by 200, 165, and 120 Myr, respectively. The divergence estimate for crown Metazoa shows a close coupling with the chosen maximum calibration, i.e., within 50 Myr of the designated maximum age in all models except those utilizing the Lakhanda Group or GOE. In addition, the error on divergence estimates is reduced as the maximum calibration approaches the body fossil first appearance datum for Metazoa: ± 94 Ma for a pre-Tonian calibration (i.e., Lakhanda Group and the GOE), ± 54 Ma for a more ancient Tonian calibration (i.e., Bitter Springs Group), ± 27 Ma for a younger Tonian calibration (i.e., Chuar Group), and ± 3 Ma for Ediacaran calibrations (i.e., Lantian and Weng’an biotas), implying increasing confidence in molecular estimates, even though only the maximum calibration has changed. Reducing the confidence in maximum calibrations within the molecular clock produces older divergence estimates and an asymmetric increase in uncertainty, resulting in a greater range of plausible older ages (table S2). Although there is notable variation in the age estimates of these basal nodes in the tree, the majority of subsequent diversification is post-Cryogenian in all our analyses.

## DISCUSSION

### The Kheseen Biota undermines Weng’an as a maximum calibration

Weng’an fossils have previously been interpreted as bilaterians (*24*), but their diagnostic bilaterian features were subsequently shown to be diagenetic artifacts rather than biological structures (*63*); the affinity of these fossils remains an open question (*5*). In contrast, later phosphatized Cambrian assemblages—including the Kuanchuanpu Fauna, South China, and Salaagol Formation, Mongolia—yield unambiguous bilaterian embryos, such as *Olivooides* and *Markuelia* (*26*–*28*). Thus, bilaterian embryos can be preserved via phosphatization, prompting interpretation of their absence from the Weng’an Biota as evidence that bilaterians had not evolved by the early Ediacaran (*11*, *22*). The Weng’an Biota could also be argued as a suitable maximum calibration on crown Metazoa (Fig. 4A), as morphological evidence from synchrotron computed tomography has indicated that none of the Weng’an fossils unambiguously represent crown group metazoans, instead supporting a holozoan affinity (*22*).

Comparisons between the Kheseen and Weng’an biotas undermine the proposed use of Weng’an as a maximum calibration for crown group Metazoa, Eumetazoa, and Bilateria (*11*). The known biodiversity of the Kheseen Biota is less than that of the Weng’an Biota, which has yielded >50 species [e.g., (*42*)], but several taxa (*Appendisphaera grandis*, *A. tenuis*, *Asterocapsoides wenganensis*, *Cavaspina basiconica*, *Knollisphaeridium maximum*, *Megasphaera inornata*, *M. ornata*, *Mengeosphaera puncticulosa*, *M. reticulata*, and *Variomargosphaeridium gracile*) are shared between the two [also see (*34*)]; *Megasphaera puncticulosa* and *Mengeosphaera reticulata* are otherwise known only from Weng’an (*42*, *43*). Although the diversity is lower (*34*), the morphological details preserved in specimens from the Kheseen Biota rival those from Weng’an and later Cambrian phosphatized deposits. The Weng’an Biota contains exceptionally well-preserved microfossils as demonstrated, for example, by the preservation of 0.5- to 0.7-μm-wide ridges on *M. reticulata* [figures 25.9 to 25.11 in (*42*)]. The polygonal fields of *Mengeosphaera* from the Kheseen Biota are delineated by ridges <1-μm wide (Fig. 2G), almost identical to those described from Weng’an (*42*), and individual processes on specimens of *Appendisphaera*, *Knollisphaeridium*, and *Mengeosphaera* are as fine as 1.5 μm [this study and (*32*)].

The equivalent fidelity of preservation in the two biotas reflects similar depositional settings with analogous taphonomic pathways. Phosphatized microfossils are confined to two units within the Kheseen Formation. The lower unit records primary phosphatization within a shallow, energetic depozone located on a tectonically mediated and sediment-starved paleotopographic high. The migration of this paleotopographic high resulted in reworking and redeposition of primary phosphatic material as allochems in grainstones of the upper unit (*35*). These depositional conditions and facies associations are directly analogous to those inferred for the granular phosphorites hosting the Weng’an Biota [figure 3C in (*64*)]. The Weng’an phosphorites formed in an outer shelf environment (*64*) evidenced by their association with stromatolites and intraclastic material, which indicate deposition within the photic zone and above fair-weather wave base (*45*). Concentrated granular phosphorite horizons host Weng’an microfossils as allochems (*45*), suggesting that phosphatization of the original organic material occurred elsewhere in the basin before the grains were reworked, concentrated, and redeposited (*64*) along a sediment-starved topographic high, represented by the outer shelf (*65*).

The Weng’an Biota is at least 25 Myr older than the Kheseen Biota (Fig. 4B). A Re-Os age constrains its maximum age to 587.2 ± 3.6 Ma (*1*), with a minimum age of 574.0 ± 4.7 Ma (*1*) provided by the presence of the Shuram (EN3) excursion above its youngest occurrence. In contrast, geochemical and geochronological data indicate that the Kheseen Formation spans the interval of the Ediacaran–Cambrian boundary (Fig. 1B). Animal life was already flourishing at this time (Fig. 4B), with the Kheseen Biota occurring, at the earliest, contemporaneously with the late Ediacaran Nama assemblage (*66*), which includes animals with biomineralized skeletons [e.g., (*67*)], crown group sponges (*68*), and body and trace fossils of motile bilaterians [e.g., (*69*)]. Small carbonaceous fossils, some of which may represent bilaterians (*70*), are also coeval with the Nama assemblage (*2*). This demonstrates that not only metazoans, but also eumetazoans and bilaterians, had evolved by the time of Kheseen Formation deposition.

The absence of animals in the Kheseen Biota (also see the Supplementary Materials), despite them having evolved by its time of deposition, calls into question the use of the Weng’an Biota as a maximum calibration for crown group Metazoa, Eumetazoa, or Bilateria. The lack of evidence for animals in the Kheseen Biota must be due to other factors, such as taphonomy or ecology, and these same factors cannot be ruled out when accounting for the lack of animals at Weng’an. For instance, the inundation of sediment/pore waters with labile phosphorus is mediated by redox sensitive processes (*35*), implying conditions that may have been inhospitable for early bilaterian animals. As the lack of animal fossils in phosphatized Ediacaran–Cambrian biotas does not necessarily imply that they had not yet evolved, such biotas are unsuitable for use as maximum calibrations.

### Challenges for alternative Ediacaran maximum calibrations

The older Lantian Biota (∼602 ± 7 Ma) (*19*) represents an alternative Ediacaran calibration (*11*, *18*) to Weng’an. Some Lantian fossils have been allied with cnidarians based on their tentacle and finger-like structures (*20*, *21*), but these comparisons rely on gross morphological similarities; convincing evidence for a cnidarian, or even metazoan, affinity is lacking. These fossils, preserved as carbonaceous compressions, may alternatively represent macroalgae, which dominate the remainder of the assemblage (*5*, *21*). If confirmed, then the Lantian Biota could be used as a maximum calibration for crown Metazoa if it were preserved via BST preservation (*20*) because it lacks unambiguous animal body fossils. However, the geochemical pathways to fossilization in the Lantian Biota are poorly constrained. Pyritization plays a role (*21*), but clays, which are known to be important in BST preservation (*71*), are not well-documented (*72*). Without geochemical evidence demonstrating BST preservation, the taphonomic pathway of the Lantian Biota, and its potential for preserving animal tissues, remains uncertain.

The younger Miaohe Biota (∼551 Ma), Dengying Formation, China, similar to the Lantian, is dominated by benthic multicellular algae in addition to morphologically simple macrofossils of uncertain affinity, none of them conclusively animal (*73*). These fossils, akin to those of the Lantian Biota, are also preserved as carbonaceous compressions, primarily through kerogenization (*74*), but pyritization and aluminosilicification may also contribute (*73*). Animals, if they were present, would likely be preserved as carbonaceous compressions within the Miaohe Biota through processes similar to BST preservation. The occurrence of unequivocal animal fossils elsewhere in South China (*75*) and worldwide [e.g., (*66*)] by 550 Ma demonstrates the existence of an established and diverse animal fauna at the time of Miaohe preservation (Fig. 4B). The Jiangchuan Biota, another latest Ediacaran (<554 Ma) South China fossil assemblage, is a prime example, preserving both algae and animals, including bilaterians (*76*). Its occurrence emphasizes that broadly contemporaneous biotas from a single geographic region preserved as carbonaceous compressions include some that are algal dominated (Miaohe) and others that include diverse metazoans (Jiangchuan). The absence of animal fossils in the Miaohe Biota must reflect other controls (i.e., ecological, taphonomic) on their occurrence rather than implying they had yet to evolve. An analogous scenario cannot therefore be ruled out for the taphonomically similar Lantian Biota.

Older evidence for the presence of animals in the preceding Cryogenian also complicates the implementation of Ediacaran maximum calibrations. Specifically, the biomarker record suggests the presence of pre-Ediacaran animals (Fig. 4B). The C_30_ steranes 24-*iso*-propylcholestane and 26-methylstigmastane have been proposed as evidence for the presence of demosponges in the Cryogenian (*3*, *46*). Trace amounts of these biomarker compounds have been shown to be produced through diagenesis or by other organisms, most notably algae and protists (*4*), but despite substantial prior controversy (*77**–**81*), there is an emerging consensus that there is sufficient abundance and diversity of biosignatures specific to sponges preserved in Cryogenian successions to support the presence of Porifera by at least 650 Ma (*46*). The recognition of the C_31_ sterols 24-*n*-butylcholestane and 24-*sec*-butylcholestane and the combination of C_31_ and C_30_ biosignatures in abundances closely resembling those found in modern demosponges support a pre-Ediacaran origin for the group (*4*). Given that it is demosponges, a derived group, that are known to produce these biomarkers, crown Porifera must have originated before ∼650 Ma (*81*).

### A prospectus for selecting reliable maximum calibrations

Determining the difference between true divergence time and the oldest fossil occurrence of a clade is challenging as, by definition, there is no fossil evidence of a clade beyond its known temporal range (*82*). To apply a maximum calibration to a molecular clock requires establishing whether the absence of a taxon from a particular stratigraphic unit is real or an artifact of incomplete fossilization. Soft bounds are applied in most molecular clock analyses based on a set nominal probability that the calibration is violated (*10*). This probability of violation is normally 2.5% [e.g., (*11*, *15*)] for calibrations of the basal nodes within animal phylogeny. This implies 97.5% confidence in the evidence for absence of early animals from the geological record, a measure that must account for uncertainties about the ecology, taphonomy, and recognizability of these organisms.

The importance of maximum calibrations for estimating the age of basal animal nodes (Fig. 4A) and the challenges of reliably determining the nature of absence demand a protocol for their selection. A maximum calibration should meet all the criteria for preserving the first fossils of a clade but lack any representative fossils (body, trace, and biomarker) (*13*) to increase the likelihood that their absence is a true signal and not an artifact of preservational bias. However, unlike the first fossil occurrence of a clade, which requires only a single unequivocal, well-dated fossil representative, we argue, in the prospectus below, that an evidence-of-absence maximum calibration requires a more extensive set of criteria to demonstrate true absence on a global scale (Fig. 5). These include:

**Fig. 5.**
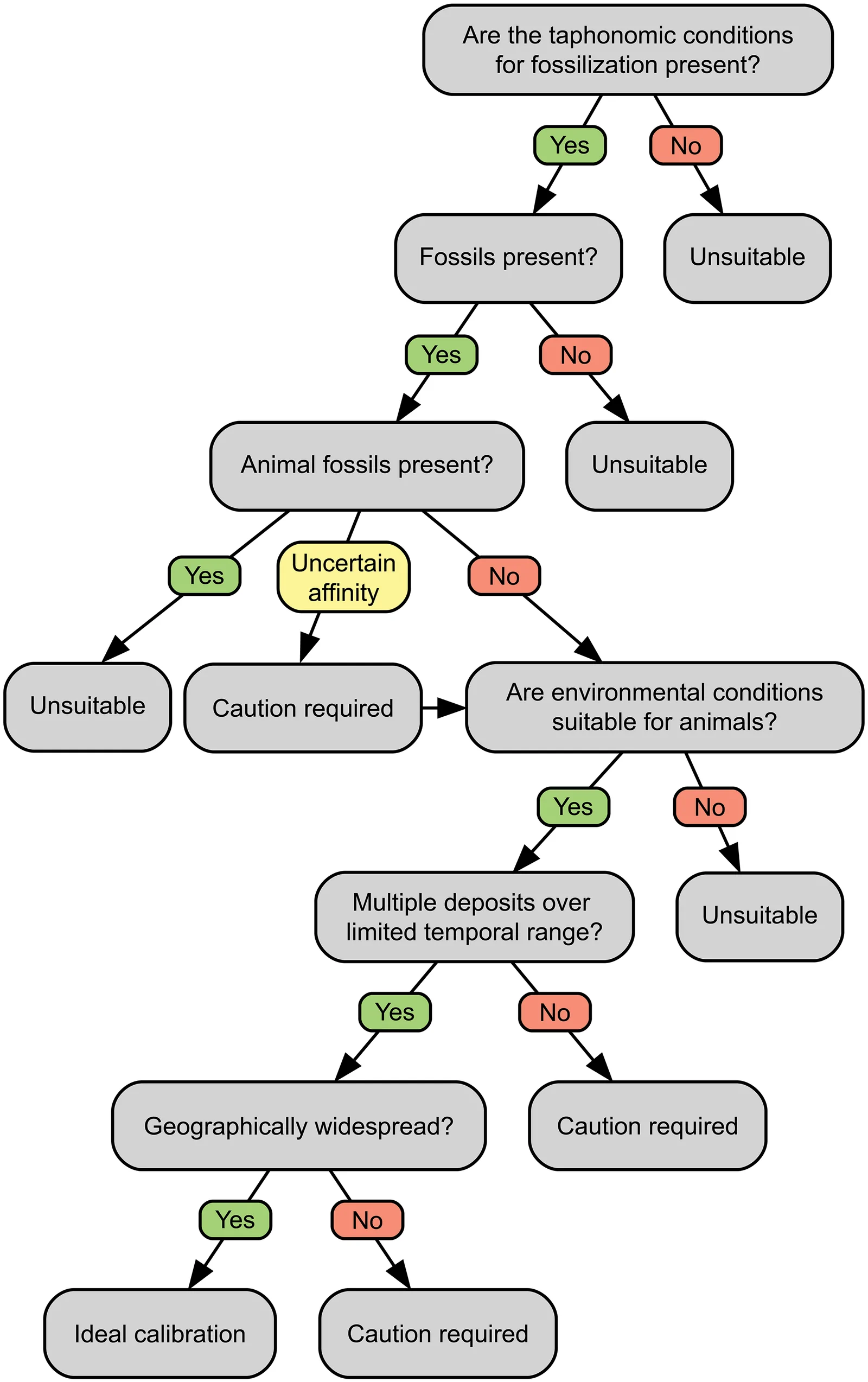
Decision tree for determining suitability of evidence-of-absence maximum calibrations on molecular clock analyses.

1) Demonstrating that appropriate geochemical conditions for the ecological success and preservation of animals are present in deposits that are candidates for maximum calibrations: Ediacaran and Cambrian body fossil assemblages, including those with the earliest animal fossils, are almost exclusively sourced from sites of exceptional soft-tissue preservation, so-called fossil Lagerstätten (*17*), and restricted to the preservational environment and style associated with each deposit. Although a number of taphonomic pathways are important during the Ediacaran and Cambrian (*83*), the earliest definitive animal communities are frequently preserved via early silica cementation in the case of the Ediacara Macrobiota (*84*), as carbonaceous compressions through BST preservation (*16*), or as a result of phosphatization (*28*). Microfossil communities typical of the Tonian, which lack animal representatives, commonly occur as kerogenized organic material, with fossilization potential enhanced by the presence of clay minerals (*85*). Some of these deposits may share taphonomic pathways with Cambrian BST preservation, known to preserve an array of animal tissues (*86*, *87*), which is likewise influenced by clay minerals (*52*). However, sites of exceptional fossil preservation may capture a restricted range of paleoenvironmental settings [e.g., (*88*)], which would potentially not overlap with those most likely to have fostered early animal communities.

Whether any of these taphonomic pathways would record evidence for the earliest, simple animals is an open question (*81*). Porifera, as the most basal animal clade, are expected to be the earliest animals represented in the fossil record. Molecular (*18*, *89*) and fossil (*68*) evidence indicate that early poriferans lacked biomineralization and were supported by an organic scaffold (*68*). Oceans at the time were likely oxygen-poor and food-limited (*81*), exerting substantial ecological and physiological constraints on early animals. Physiological calculations suggest that these conditions would favor small, thin body plans (*90*), a pattern supported by observations of organisms inhabiting oxygen minimum zones today (*91*). Nematodes, which are abundant in such modern ocean settings, provide a useful analog for early animals (*81*). Their marine fossil record is especially poor (*92*); the oldest examples occur in the terrestrial Rhynie Chert, Scotland, where they are preserved via silicification (*93*).

Despite this uncertainty, the demonstration of geochemical conditions for at least one type of exceptional preservation favoring soft-bodied animal preservation in a candidate deposit for a maximum calibration is a starting point for confirming the absence of animals of pre-Ediacaran age (*94*).

2) Demonstrating the absence of all types of fossil evidence: Advances in documenting and interpreting the biomarker record [e.g., (*4*)] allow the absence of animal biomarkers (chemical fossils) to be used alongside the absence of body and trace fossils.

3) Avoiding the use of deposits with fossils of plausible but unconfirmed animal affinity: The earliest animals are difficult to differentiate from their holozoan ancestors (*95*). Consequently, the nature of many putative early animal fossils is debated [e.g., whether Doushantuo microfossils represent animal embryos or those of non-metazoan holozoans (*5*, *22*, *23*)]. Deposits that include even putative animals should not be used as maximum calibrations until the affinities of their fossils can be clarified without controversy.

4) Demonstrating that evidence-of-absence is global: A plausible global absence of a clade necessitates consideration of multiple deposits spanning a narrow temporal range. Although only a single maximum calibration age can be selected, evidence from independent deposits of similar age can support a chosen calibration. Each occurrence must show ecological and geological conditions favoring the presence and preservation of animals, but nonetheless yield no animal fossils. Incorporating multiple geographically disparate deposits reduces sampling bias, improves representation of global diversity, and increases paleoenvironmental representation. Paleogeographic reconstructions indicate substantial geographical variation in preservational settings during the late Ediacaran (*96*), and biomarker distributions for the Ediacaran (*97*) and Tonian (*57*) reveal substantial biogeochemical heterogeneity in the oceans, emphasizing the need for evidence from numerous deposits to confirm the global absence of animals.

### The most reliable current maximum calibrations

Although the oldest definitive macroscopic animal body fossils are dated to ∼574 Ma (*1*), lipid biomarkers likely push the minimum age of crown Metazoa back to ∼650 Ma (*46*). However, almost no deposits that have been proposed as maximum calibrations beyond ∼650 Ma (*6*–*9*, *14*, *15*) fulfill all the criteria outlined in our prospectus above. For these calibrations, there is a certain degree of ambiguity as to whether they meet individual criteria or not (e.g., we do not know precisely the mechanisms of fossilization in some deposits, nor have we fully understood the taphonomic conditions that lead to animal soft-tissue fossilization or its geochemical fingerprints). This may not rule out their use as calibrations, but evidence-of-absence at a 97.5% threshold is questionable.

Perhaps the only maximum calibration for Metazoa that could be employed with the aforementioned level of confidence is the GOE at ∼2400 to 2300 Ma. Multiple lines of geological and geochemical evidence document a substantial rise in atmospheric oxygen globally in differing paleoenvironments at this time (*62*). The presence of detrital pyrite and uraninite indicate that pO_2_ was less than 1.6 × 10^−4^ of present atmospheric levels (PAL) before the GOE (*98*). As animals are estimated to have required at least 0.5% PAL (*60*), they cannot have originated before ∼2300 Ma. Using the GOE as a maximum calibration results in almost identical divergence times and uncertainties to those yielded when using the ∼1000 Ma Lakhanda Group as a maximum calibration (table S2). This suggests that a combination of the molecular data used by the clock model (see Materials and Methods) and more well-constrained nodes [e.g., Crown Gastropoda, calibrated 531.5 to 470.2 Ma (*11*)] preclude a pre-Tonian origination of animals.

Ruling out Ediacaran deposits as suitable maximum calibrations, Cryogenian and Tonian deposits should be the focus of future analyses. A variety of late Tonian (850 to 730 Ma) deposits (Fig. 4B) are perhaps the best current candidate maximum calibrations. Geochemical data show that the Svanbergfjellet and Wynniatt (Canada) formations preserve microfossils via BST preservation (i.e., clay templating) (*94*), which is known to be important in the Ediacaran and Cambrian (*16*), while the Bitter Springs Group fossils are a product of silicification, a taphonomic pathway that preserves even the most delicate animals to a high fidelity (*93*). The Chuar Group, which is likewise highly fossiliferous and lacks animals, preserves microfossils, likely through sulfurization of original organic matter (*99*), alongside nonanimal lipid biomarkers (*57*). However, there remains uncertainty about the implementation of Tonian deposits as maximum calibrations for the origin of animals. The evidence supporting their use falls far from the ideal situation, outlined above, where evidence from many geographically disparate deposits, encompassing various depositional environments and taphonomic processes, is available to confirm the absence of animals globally. Similarly, the low probability of early animal preservation (*81*, *94*) allowing confident identification remains a barrier for the utilization of these Tonian assemblages as maximum calibrations.

Paleontological and geochemical data documenting the taxonomy and taphonomy of known and new assemblages (body, trace, and biomarkers) from other sites globally in the 850- to 700-Ma interval [e.g., Visingsö, Sweden (*100*)] could bolster confidence in a late Tonian maximum calibration. Until such data are obtained, one avenue available to acknowledge the uncertainty in Tonian calibrations is to reduce the confidence in such a calibration within the molecular clock analysis. Using a higher probability of violation upon a suitable Tonian calibration—such as the Bitter Springs Group, Svanbergfjellet Formation, or Chuar Group—for example, 30% (Fig. 6), perhaps offers the best candidate for a maximum calibration at present.

**Fig. 6.**
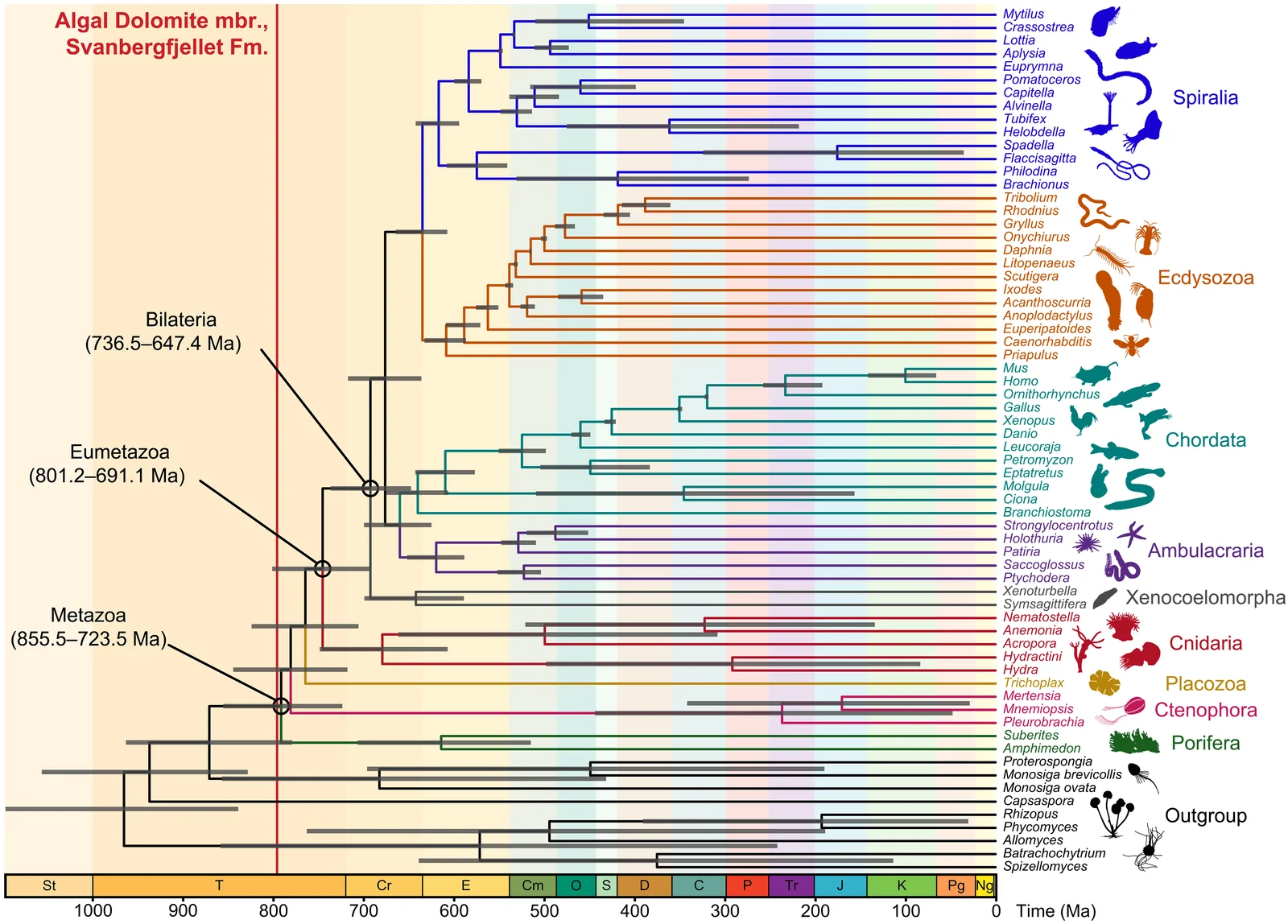
An example of divergence estimates for the animal clade using a Tonian (Algal Dolomite member, Svanbergfjellet Formation, in this instance) maximum calibration (vertical red line) with 70% confidence. Nodes of interest are labeled. The 95% HPD intervals are indicated by gray bars.

### A revived pre-Ediacaran history of animal evolution?

Discounting the recently proposed Weng’an and Lantian Biota maximum calibrations revives the possibility, previously suggested [e.g., (*14*, *15*)], that animal diversification extends into the Tonian (Fig. 4A). However, the dependence of the animal evolutionary timescale on an uncertain maximum calibration limits our ability to assess potential evolutionary drivers behind their evolution, such as overcoming intrinsic genetic challenges, environmental perturbations (e.g., a rise in oxygen concentrations or increased oceanic nutrient delivery), or ecological developments (*81*). For instance, the possibility of a pre-Ediacaran origin and diversification of animals means we cannot discount hypotheses that link the timing of animal diversification to Cryogenian Snowball Earth glaciations (*101*, *102*). The causes and trajectory of animal evolution will remain uncertain until paleontological and geochemical data provide sufficient confidence in maximum calibrations to ensure a reliable timescale.

## MATERIALS AND METHODS

Eight samples from Sections M602 and M603 [Kheseen Gol; (*38*)] were analyzed for strontium isotope (^87^Sr/^86^Sr) chemostratigraphy. Micritic carbonate hand samples were selected from exposures with minimal visible evidence of post-depositional alteration at each measured section. Hand samples were cut orthogonal to bedding, screened for textural evidence of alteration, and drilled along bedding planes with a dental burr to procure ∼50 mg of rock powder. Following several washing steps to remove clays and other terrigenous particulate material, the carbonate powders were dissolved in acetic acid, refluxed with nitric acid, and the resultant solution passed through a series of columns to isolate Sr [following (*39*)]. Sr isotopes were subsequently measured on a Thermo Fisher Scientific Neptune multicollector inductively coupled plasma mass spectrometer (MC-ICP-MS) at Woods Hole Oceanographic Institute (WHOI), Woods Hole, MA, USA, and corrected for Kr and Rb mass biases sensu (*39*). Reduced ^87^Sr/^86^Sr ratios and section locations are tabulated in table S1. δ^13^C chemostratigraphic data from sections M602 and M603 were previously integrated into a composite Khuvsgul Group δ^13^C curve (*35*), allowing the ^87^Sr/^86^Sr data collected from the same sections to be directly integrated into the Khuvsgul chemostratigraphic framework (Fig. 1B). Similarly, δ^13^C and ^87^Sr/^86^Sr data from other Khuvsgul Group localities (*103*) were integrated into the framework by correlating δ^13^C chemostratigraphy of individual sections with the composite Khuvsgul Group δ^13^C curve of (*35*)*.*

Microfossils were recovered from the Kheseen Formation at six localities, sampled as nine stratigraphic sections along the western shore of Lake Khuvsgul (Fig. 1A and table S3). Specimens were identified in polished petrographic thin sections (30-μm thickness oriented perpendicular to bedding, *n* = 233 thin sections from 143 samples) using a Zeiss Axioscope A1 microscope with an Axiocam 105 camera at the Museum of Natural History, University of Oxford, Oxford, UK. A subset of samples (*n* = 117, 110 of which were also examined in thin section) were dissolved in 20% acetic acid. Samples (*n* = 100) yielded acid-insoluble phosphatic microfossils that were dried at 50°C and mounted on scanning electron microscope (SEM) stubs using carbon tape. Stubs were gold-coated and examined using a Quanta FEG 650 SEM at 10 kV at the Department of Earth Sciences, University of Oxford, Oxford, UK and a Hitachi SU7000 SEM at 10 kV at the Department of Earth and Planetary Sciences, Yale University, New Haven, CT, USA.

We assessed the sensitivity of molecular clocks to the choice of maximum calibrations on early animal evolution to determine how calibration selection influences divergence estimates. A recent molecular clock for the evolutionary history of animals (*11*) was selected as a base model, with maximum and minimum calibrations varied. Table S2 provides a summary of the 13 models run, detailing the combinations of calibrations used and the resulting divergence estimates for Metazoa, Eumetazoa, and Bilateria. Variants were run to explore any differences resulting from the choice of minimum calibration. The standard tree of (*11*)—with Porifera as sister to all other animals, Xenacoelomorpha as sister to Nephrozoa, and monophyletic Deuterostomia—was selected as the base topology for our analyses. An autocorrelated clock model calibrated using a uniform distribution scheme, with a 0.1% soft minimum bound and a 2.5% soft maximum bound (except where otherwise stated, table S2), was used according to sensitivity analyses conducted by (*11*). The uniform distribution scheme represents a median interpretation of fossil record reliability, incorporating the least associated uncertainty in calibrations. Analyses were performed using MCMCtree v4.10.9 (*104*), with an approximate likelihood calculation and the LG + G model of amino acid substitution (*105*). The prior for the rate variance (σ2) was set to G (1, 10), a gamma distribution with shape of 1 and scale of 10, allowing serious violation of the clock (*14*). Analyses were run for 20,000 iterations after a burn-in of 100,000 with a sampling frequency of 1000. In all analyses, the maxima of nodes calibrated using the ages of the Weng’an (590.8 Ma) or Lantian (609.0 Ma) biotas [for full list of affected nodes, see (*11*)] and the minima of Metazoa, Eumetazoa, and Bilateria were updated to reflect alternative calibration schemes; all other input data and model parameters were unchanged from (*11*).

## Supplementary Materials

### The PDF file includes:

Tables S3 and S4 Supplementary Discussion of Purported Kheseen SSFs Provenance statement for fossil material Legends for tables S1 and S2 References
