# Supplementary material for: Re-evaluating molecular clock maximum age calibrations revives pre-Ediacaran divergence estimates for animals
Source: Sci Adv. 2026 Oct 2;12(40):eaeg6289. doi: 10.1126/sciadv.aeg6289 (PMC13632735; doi:10.1126/sciadv.aeg6289)
Supplement: Supplementary file 1 [file sciadv.aeg6289_sm.pdf]

Science Advances
AAAS

Supplementary Materials for

Re-evaluating molecular clock maximum age calibrations revives pre-Ediacaran divergence estimates for animals

Orin M. Lole Durbin *et al.*

Corresponding author: Orin M. Lole Durbin, orinloledurbin@vt.edu;
Ross P. Anderson, ross.anderson@oum.ox.ac.uk

Sci. Adv. **12**, eaeg6289 (2026)
DOI: 10.1126/sciadv.aeg6289

The PDF file includes:

Tables S3 and S4
Supplementary Discussion of Purported Kheseen SSFs
Provenance statement for fossil material
Legends for tables S1 and S2
References

Other Supplementary Material for this manuscript includes the following:

Tables S1 and S2

Sample Label	Location	Coordinates	Sample Type	Samples collected	Thin sections	Dissolved Samples	Fossiliferous Samples
**EA1701**	Bakha Gol	50°52'32.64"N, 100°10'46.07"E	Subcrop	6	6	3	2
**EA1704**	Kheseen Gol	50°44'45.49"N, 100°11'25.67"E	*In situ*	38	31	13	8
**EA1705**	Kheseen Gol	50°44'49.32"N, 100°11'31.57"E	*In situ*	39	33	15	6
**RPA1701**	Nurga Gol	50°52'13.58"N, 100°13'19.36"E	Float	35	35	26	4
**RPA1702**	Nurga Gol	50°52'11.49"N, 100°13'5.04"E	Subcrop and float	61	60	40	37
**F1703**	South of Nurga Gol ridge	50°51'5.73"N, 100°11'49.86"E	Subcrop and float	18	17	6	3
**F1704**	South of Nurga Gol ridge	50°51'5.73"N, 100°11'49.86"E	Subcrop and float	32	32	8	1
**F1707**	Ikh Mountain	50°42'12.49"N, 100°07'17.14"E	*In situ*	4	4	2	1
**JP1717**	Khirvesteg Gol	50°41'32.07"N, 100°10'14.57"E	*In situ* and subcrop	20	15	4	1

Table S3: Summary of sampled localities on the western bank of Lake Khuvsgul. Description of terms: *In situ* = situated in original position; Subcrop = eroded material situated in presumed near original position; Float = loose rock transported away from original position.

	EA1701	EA1704	EA1705	RPA1701	RPA1702	F1703	F1704	F1707	JP1717	M602 176.0	M602 179.0	M618 32.0	M618 33.0
Appendisphaera grandis		R										R	
Appendisphaera fragilis											R		R
Appendisphaera tenuis		R										R	
Appendisphaera **sp.**					R								
Cavaspina basiconica												R	
Asterocapsoides wenganensis			R		R								
Knollisphaeridium maximum			R		R								
Leiosphaeridia **spp.**	R	C	C	R	C			R		R	R	C	C
Megasphaera inornata		C	R	R	C								
Megasphaera ornata					R								
Megasphaera minuscula		R		R	R							C	C
Megasphaera ?puncticulosa												R	
Mengeosphaera reticulata		R	R										
Sinosphaera **sp.**			R										
Variomargosphaeridium aculeiparvum													R
Variomargosphaeridium gracile												C	C
Variomargosphaeridium **sp.**					R								

Table S4: Kheseen Formation microfossil occurrences. Taxa are reported from the nine sampled localities along the western shore of Lake Khuvsgul investigated during this study and their relative abundance within the assemblage is indicated. Also shown (grey shade) is the reported occurrence data for previous investigations *(31, 32)*. See *(32)* for sample localities. R = rare (isolated individuals, only a few specimens). C = common (10s of individuals). For microfossil occurrences to the south of Lake Khuvsgul, see *(34)*.

Discussion of purported Kheseen SSF Fossils

SSFs and archaeocyath fragments, representing animals, have been reported from drill core of the Kheseen Formation *(107)*, but the stratigraphic position of this material is uncertain. The SSFs were reported from diamictite, a lithology not recognized in recent stratigraphic assessments of the Kheseen Formation *(30, 35)*. The sampled drill core intersected an inferred fault zone [*(107)*, fig. 2] and the material may derive from fault gouge breccia previously misinterpreted as diamictite. Subsequent investigations have recovered neither SSFs nor archaeocyath fragments from Kheseen Formation phosphorites *(31, 32, 34,* this study*)*, but archaeocyaths are known from the Middle Member of the overlying Erkhelnuur Formation (Fig. 1b). Thus, the breccia infilling the fault zone, the source of the SSFs and archaeocyath fragments previously reported *(107)*, most likely represents material from the Erkhelnuur Formation.

Provenance statement

Fossil material described in this study was recovered from the Kheseen Formation exposed to the west of Lake Khuvsgul, Mongolia. Field collection was conducted by Ross Anderson, Eliel Anttila, and Francis Macdonald in 2017 in collaboration with the Mongolian University of Science and Technology and complied with all applicable regulations. All fossil material was exported, imported, and studied in accordance with applicable local, national, and international laws. A justification for a late Ediacaran/early Cambrian age for the fossils is provided in the manuscript that accompanies these supplementary materials. All microfossils recovered in this work are reposited in the Invertebrate Paleontology Division of the Yale Peabody Museum and are available for study.

Table S1: Isotope data for Kheseen Formation sections M602 and M603. $\delta^{13}C$ (VPDB), $\delta^{18}O$ (VPDB), and $^{87}Sr/^{86}Sr$ are presented for samples (indicated by stratigraphic height) from each of the two sections. Note that italicized data are outside stratigraphic extent of plots in Fig. 1B.

Table S2: Summary of molecular clock model variations run during calibration impact analyses. Calibration ages, calibration justification, sources, and results are provided for each variant.

REFERENCES

1. C. Yang, A. D. Rooney, D. J. Condon, X.-H. Li, D. V. Grazhdankin, F. T. Bowyer, C. Hu, F. A. Macdonald, M. Zhu, The tempo of Ediacaran evolution. *Sci. Adv.* **7**, eabi9643 (2021).

2. B. J. Slater, M. S. Bohlin, Animal origins: The record from organic microfossils. *Earth Sci. Rev.* **232**, 104107 (2022).

3. G. D. Love, E. Grosjean, C. Stalvies, D. A. Fike, J. P. Grotzinger, A. S. Bradley, A. E. Kelly, M. Bhatia, W. Meredith, C. E. Snape, S. A. Bowring, D. J. Condon, R. E. Summons, Fossil steroids record the appearance of Demospongiae during the Cryogenian period. *Nature* **457**, 718–721 (2009).

4. L. Shawar, G. D. Love, B. T. Uveges, J. A. Zumberge, P. Cárdenas, J.-L. Giner, R. E. Summons, Chemical characterization of C31 sterols from sponges and Neoproterozoic fossil sterane counterparts. *Proc. Natl. Acad. Sci. U.S.A.* **122**, e2503009122 (2025).

5. J. A. Cunningham, A. G. Liu, S. Bengtson, P. C. J. Donoghue, The origin of animals: Can molecular clocks and the fossil record be reconciled? *Bioessays* **39**, 1–12 (2017).

6. K. J. Peterson, J. A. Cotton, J. G. Gehling, D. Pisani, The Ediacaran emergence of bilaterians: Congruence between the genetic and the geological fossil records. *Philos. Trans. R. Soc. B Biol. Sci.* **363**, 1435–1443 (2008).

7. E. A. Sperling, J. M. Robinson, D. Pisani, K. J. Peterson, Where's the glass? Biomarkers, molecular clocks, and microRNAs suggest a 200-Myr missing Precambrian fossil record of siliceous sponge spicules. *Geobiology* **8**, 24–36 (2010).

8. D. H. Erwin, M. Laflamme, S. M. Tweedt, E. A. Sperling, D. Pisani, K. J. Peterson, The Cambrian conundrum: Early divergence and later ecological success in the early history of animals. *Science* **334**, 1091–1097 (2011).

9. M. Dohrmann, G. Wörheide, Dating early animal evolution using phylogenomic data. *Sci. Rep.* **7**, 3599 (2017).

10. M. J. Benton, P. C. J. Donoghue, Paleontological evidence to date the tree of life. *Mol. Biol. Evol.* **24**, 26–53 (2007).

11. E. Carlisle, Z. Yin, D. Pisani, P. C. J. Donoghue, Ediacaran origin and Ediacaran-Cambrian diversification of Metazoa. *Sci. Adv.* **10**, eadp7161 (2024).

12. C. R. Marshall, Using the fossil record to evaluate timetree timescales. *Front. Genet.* **10**, 1049 (2019).

13. J. F. Parham, P. C. J. Donoghue, C. J. Bell, T. D. Calway, J. J. Head, P. A. Holroyd, J. G. Inoue, R. B. Irmis, W. G. Joyce, D. T. Ksepka, J. S. L. Patané, N. D. Smith, J. E. Tarver, M. van Tuinen, Z. Yang, K. D. Angielczyk, J. M. Greenwood, C. A. Hipsley, L. Jacobs, P. J. Makovicky, J. Müller, K. T. Smith, J. M. Theodor, R. C. M. Warnock, M. J. Benton, Best practices for justifying fossil calibrations. *Syst. Biol.* **61**, 346–359 (2012).

14. M. Dos Reis, Y. Thawornwattana, K. Angelis, M. J. Telford, P. C. J. Donoghue, Z. Yang, Uncertainty in the timing of origin of animals and the limits of precision in molecular timescales. *Curr. Biol.* **25**, 2939–2950 (2015).

15. H. C. Betts, M. N. Puttick, J. W. Clark, T. A. Williams, P. C. J. Donoghue, D. Pisani, Integrated genomic and fossil evidence illuminates life's early evolution and eukaryote origin. *Nat. Ecol. Evol.* **2**, 1556–1562 (2018).

16. R. R. Gaines, Burgess Shale-type preservation and its distribution in space and time. *Paleontol. Soc. Pap.* **20**, 123–146 (2014).

17. R. R. Gaines, M. L. Droser, Fossil Lagerstätten and the enigma of anactualistic fossil preservation. *Paleobiology* **51**, 29–43 (2025).

18. M. E. Rossi, J. N. Keating, N. J. Kenny, M. Giacomelli, S. Álvarez-Carretero, A. Schuster, P. Cárdenas, S. Taboada, V. Koutsouveli, P. C. J. Donoghue, A. Riesgo, D. Pisani, Independent origins of spicules reconcile paleontological and molecular evidence of sponge evolutionary history. *Sci. Adv.* **12**, eadx1754 (2026).

19. C. Yang, Y. Li, D. Selby, B. Wan, C. Guan, C. Zhou, X.-H. Li, Implications for Ediacaran biological evolution from the ca. 602 Ma Lantian biota in China. *Geology* **50**, 562–566 (2022).

20. X. Yuan, Z. Chen, S. Xiao, C. Zhou, H. Hua, An early Ediacaran assemblage of macroscopic and morphologically differentiated eukaryotes. *Nature* **470**, 390–393 (2011).

21. B. Wan, X. Yuan, Z. Chen, C. Guan, K. Pang, Q. Tang, S. Xiao, Systematic description of putative animal fossils from the early Ediacaran Lantian Formation of South China. *Palaeontology* **59**, 515–532 (2016).

22. W. Sun, Z. Yin, P. Liu, M. Zhu, P. C. J. Donoghue, Developmental biology of *Spiralicellula* and the Ediacaran origin of crown metazoans. *Proc. R. Soc. B Biol. Sci.* **291**, 20240101 (2024).

23. S. Xiao, Y. Zhang, A. H. Knoll, Three-dimensional preservation of algae and animal embryos in a Neoproterozoic phosphorite. *Nature* **391**, 553–558 (1998).

24. J.-Y. Chen, D. J. Bottjer, G. Li, M. G. Hadfield, F. Gao, A. R. Cameron, C.-Y. Zhang, D.-C. Xian, P. Tafforeau, X. Liao, Z.-J. Yin, Complex embryos displaying bilaterian characters from Precambrian Doushantuo phosphate deposits, Weng'an, Guizhou, China. *Proc. Natl. Acad. Sci. U.S.A.* **106**, 19056–19060 (2009).

25. Z. Yin, M. Zhu, D. J. Bottjer, F. Zhao, P. Tafforeau, Meroblastic cleavage identifies some Ediacaran Doushantuo (China) embryo-like fossils as metazoans. *Geology* **44**, 735–738 (2016).

26. X.-P. Dong, S. Bengtson, N. J. Gostling, J. A. Cunningham, T. H. P. Harvey, A. Kouchinsky, A. K. Val'kov, J. E. Repetski, M. Stampanoni, F. Marone, P. C. J. Donoghue, The anatomy, taphonomy, taxonomy and systematic affinity of *Markuelia*: Early Cambrian to Early Ordovician scalidophorans. *Palaeontology* **53**, 1291–1314 (2010).

27. X. P. Dong, K. Vargas, J. A. Cunningham, H. Zhang, T. Liu, F. Chen, J. Liu, S. Bengtson, P. C. J. Donoghue, Developmental biology of the Early Cambrian cnidarian *Olivooides*. *Palaeontology* **59**, 387–407 (2016).

28. M. Steiner, B. Yang, S. Hohl, D. Li, P. C. J. Donoghue, Exceptionally preserved early Cambrian bilaterian developmental stages from Mongolia. *Nat. Commun.* **12**, 1037 (2021).

29. J. P. T. Hippertt, I. D. Rudnitzki, L. Morais, B. T. Freitas, G. R. Romero, M. Babinski, M. G. Leite, J. M. Leme, H. A. Nalini, L. B. Nogueira, R. I. F. Trindade, Assessing chemostratigraphic and biostratigraphic correlation of Ediacaran phosphorites (Bocaina, Khesen and Doushantuo Formations): Diachronic, local signals of the Neoproterozoic phosphogenic-taphonomic event. *Precambrian Res.* **405**, 107383 (2024).

30. E. S. Anttila, F. Macdonald, U. Bold, Stratigraphy of the Khuvsgul Group, Mongolia. *Mong. Geosci.* **26**, 2–15 (2021).

31. R. P. Anderson, F. A. Macdonald, D. S. Jones, S. McMahon, D. E. G. Briggs, Doushantuo-type microfossils from latest Ediacaran phosphorites of northern Mongolia. *Geology* **45**, 1079–1082 (2017).

32. R. P. Anderson, S. McMahon, F. A. Macdonald, D. S. Jones, D. E. G. Briggs, Palaeobiology of latest Ediacaran phosphorites from the upper Khesen Formation, Khuvsgul Group, northern Mongolia. *J. Syst. Palaeontol.* **17**, 501–532 (2019).

33. F. T. Bowyer, A. Y. Zhuravlev, R. Wood, G. A. Shields, Y. Zhou, A. Curtis, S. W. Poulton, D. J. Condon, C. Yang, M. Zhu, Calibrating the temporal and spatial dynamics of the Ediacaran–Cambrian radiation of animals. *Earth Sci. Rev.* **225**, 103913 (2022).

34. X. Shang, P. Liu, D. Zhang, Long-lived Doushantuo-Pertatataka acritarchs during the Ediacaran Shuram carbon isotope excursion: Evidence from the Kheseen Formation in northwest Mongolia. *Geol. Soc. Am. Bull.* 10.1130/B38498.1 (2026).

35. E. S. C. Anttila, F. A. Macdonald, B. Schoene, S. P. Gaynor, Cambrian foreland phosphogenesis in the Khuvsgul Basin of Mongolia. *Am. J. Sci.* **325**, 3 (2025).

36. I. A. Vishnevskaya, E. F. Letnikova, Chemostratigraphy of the Vendian–Cambrian carbonate sedimentary cover of the Tuva-Mongolian microcontinent. *Russ. Geol. Geophys.* **54**, 567–586 (2013).

37. L. B. Cherry, G. J. Gilleaudeau, D. V. Grazhdankin, S. J. Romaniello, A. J. Martin, A. J. Kaufman, A diverse Ediacara assemblage survived under low-oxygen conditions. *Nat. Commun.* **13**, 7306 (2022).

38. F. A. Macdonald, D. S. Jones, Chapter 30 The Khubsugul Group, Northern Mongolia. *Geol. Soc. Lond. Mem.* **36**, 339–345 (2011).

39. U. Bold, E. F. Smith, A. D. Rooney, S. A. Bowring, R. Buchwaldt, F. O. Dudás, J. Ramezani, J. L. Crowley, D. P. Schrag, F. A. Macdonald, Neoproterozoic stratigraphy of the Zavkhan Terrane of Mongolia: The backbone for Cryogenian and Early Ediacaran chemostratigraphic records. *Am. J. Sci.* **316**, 1–63 (2016).

40. M. D. Brasier, G. Shields, V. N. Kuleshov, E. A. Zhegallo, Integrated chemo- and biostratigraphic calibration of early animal evolution: Neoproterozoic–early Cambrian of southwest Mongolia. *Geol. Mag.* **133**, 445–485 (1996).

41. E. F. Smith, F. A. Macdonald, T. A. Petach, U. Bold, D. P. Schrag, Integrated stratigraphic, geochemical, and paleontological late Ediacaran to early Cambrian records from southwestern Mongolia. *Geol. Soc. Am. Bull.* **128**, 442–468 (2016).

42. S. Xiao, C. Zhou, P. Liu, D. Wang, X. Yuan, Phosphatized acanthomorphic acritarchs and related microfossils from the Ediacaran Doushantuo Formation at Weng’an (South China) and their implications for biostratigraphic correlation. *J. Paleontol.* **88**, 1–67 (2014).

43. X. Shang, P. Liu, M. Moczydłowska, Acritarchs from the Doushantuo Formation at Liujing section in Songlin area of Guizhou Province, South China: Implications for early–middle Ediacaran biostratigraphy. *Precambrian Res.* **334**, 105453 (2019).

44. P. J. Liu, S. Xiao, C. Y. Yin, S. M. Chen, C. M. Zhou, M. Li, Ediacaran acanthomorphic acritarchs and other microfossils from chert nodules of the Upper Doushantuo Formation in the Yangtze Gorges area, South China. *J. Paleontol.* **88**, 1–139 (2014).

45. S. Xiao, A. H. Knoll, Phosphatized animal embryos from the Neoproterozoic Doushantuo Formation at Weng'an, Guizhou, South China. *J. Paleontol.* **74**, 767–788 (2000).

46. J. A. Zumberge, G. D. Love, P. Cárdenas, E. A. Sperling, S. Gunasekera, M. Rohrssen, E. Grosjean, J. P. Grotzinger, R. E. Summons, Demosponge steroid biomarker 26-methylstigmastane provides evidence for Neoproterozoic animals. *Nat. Ecol. Evol.* **2**, 1709–1714 (2018).

47. F. S. Dunn, P. C. J. Donoghue, A. G. Liu, Morphogenesis of *Fractofusus andersoni* and the nature of early animal development. *Nat. Commun.* **16**, 3439 (2025).

48. S. D. Evans, I. V. Hughes, J. G. Gehling, M. L. Droser, Discovery of the oldest bilaterian from the Ediacaran of South Australia. *Proc. Natl. Acad. Sci. U.S.A.* **117**, 7845–7850 (2020).

49. S. D. Evans, E. A. Sperling, K. V. Lau, J. V. Strauss, Discovery of White Sea assemblage fossils from Laurentia. *Sci. Adv.* **12**, eaed9916 (2026).

50. A. D. Rooney, M. D. Cantine, K. D. Bergmann, I. Gómez-Pérez, B. A. Baloushi, T. H. Boag, J. F. Busch, E. A. Sperling, J. V. Strauss, Calibrating the coevolution of Ediacaran life and environment. *Proc. Natl. Acad. Sci. U.S.A.* **117**, 16824–16830 (2020).

51. T. Zhang, C. B. Keller, M. J. Hoggard, A. D. Rooney, G. P. Halverson, K. D. Bergmann, J. L. Crowley, J. V. Strauss, A Bayesian framework for subsidence modeling in sedimentary basins: A case study of the Tonian Akademikerbreen Group of Svalbard, Norway. *Earth Planet. Sci. Lett.* **620**, 118317 (2023).

52. R. P. Anderson, N. J. Tosca, G. Cinque, M. D. Frogley, I. Lekkas, A. Akey, G. M. Hughes, K. D. Bergmann, A. H. Knoll, D. E. G. Briggs, Aluminosilicate haloes preserve complex life approximately 800 million years ago. *Interface Focus* **10**, 20200011 (2020).

53. S. M. Porter, A. H. Knoll, Testate amoebae in the Neoproterozoic Era: Evidence from vase-shaped microfossils in the Chuar Group, Grand Canyon. *Paleobiology* **26**, 360–385 (2000).

54. S. M. Porter, R. Meisterfeld, A. H. Knoll, Vase-shaped microfossils from the Neoproterozoic Chuar Group, Grand Canyon: A classification guided by modern testate amoebae. *J. Paleontol.* **77**, 409–429 (2003).

55. A. D. Rooney, J. Austermann, E. F. Smith, Y. Li, D. Selby, C. M. Dehler, M. D. Schmitz, K. E. Karlstrom, F. A. Macdonald, Coupled Re-Os and U-Pb geochronology of the Tonian Chuar Group, Grand Canyon. *GSA Bull.* **130**, 1085–1098 (2018).

56. R. E. Summons, S. C. Brassell, G. Eglinton, E. Evans, R. J. Horodyski, N. Robinson, D. M. Ward, Distinctive hydrocarbon biomarkers from fossiliferous sediment of the late Proterozoic Walcott member, Chuar Group, Grand Canyon, Arizona. *Geochim. Cosmochim. Acta* **52**, 2625–2637 (1988).

57. J. A. Zumberge, D. Rocher, G. D. Love, Free and kerogen-bound biomarkers from late Tonian sedimentary rocks record abundant eukaryotes in mid-Neoproterozoic marine communities. *Geobiology* **18**, 326–347 (2020).

58. J. J. Brocks, A. J. M. Jarrett, E. Sirantoine, F. Kenig, M. Moczydłowska, S. Porter, J. Hope, Early sponges and toxic protists: Possible sources of cryostane, an age diagnostic biomarker antedating Sturtian Snowball Earth. *Geobiology* **14**, 129–149 (2016).

59. T. N. Hermann, *Organic World Billion Year Ago*, A. V. Sochava, Ed. (Nauka, 1990), pp. 49.

60. D. B. Mills, L. M. Ward, C. Jones, B. Sweeten, M. Forth, A. H. Treusch, D. E. Canfield, Oxygen requirements of the earliest animals. *Proc. Natl. Acad. Sci. U.S.A.* **111**, 4168–4172 (2014).

61. J.-P. Duda, H. König, M. Reinhardt, J. Shuvalova, P. Parkhaev, Molecular fossils within bitumens and kerogens from the ~1 Ga Lakhanda Lagerstätte (Siberia, Russia) and their significance for understanding early eukaryote evolution. *PalZ* **95**, 577–592 (2021).

62. T. W. Lyons, C. T. Reinhard, N. J. Planavsky, The rise of oxygen in Earth's early ocean and atmosphere. *Nature* **506**, 307–315 (2014).

63. J. A. Cunningham, C.-W. Thomas, S. Bengtson, S. L. Kearns, S. Xiao, F. Marone, M. Stampanoni, P. C. J. Donoghue, Distinguishing geology from biology in the Ediacaran Doushantuo biota relaxes constraints on the timing of the origin of bilaterians. *Proc. R. Soc. B Biol. Sci.* **279**, 2369–2376 (2012).

64. S. Xiao, A. D. Muscente, L. Chen, C. Zhou, J. D. Schiffbauer, A. D. Wood, N. F. Polys, X. Yuan, The Weng'an biota and the Ediacaran radiation of multicellular eukaryotes. *Natl. Sci. Rev.* **1**, 498–520 (2014).

65. A. D. Muscente, A. D. Hawkins, S. Xiao, Fossil preservation through phosphatization and silicification in the Ediacaran Doushantuo Formation (South China): A comparative synthesis. *Palaeogeogr. Palaeoclimatol. Palaeoecol.* **434**, 46–62 (2015).

66. R. Wood, F. T. Bowyer, R. Alexander, M. Yilales, C.-I. Uahengo, K. Kaputuaza, J. Ndeunyema, A. Curtis, New Ediacaran biota from the oldest Nama Group, Namibia (Tsaus Mountains), and re-definition of the Nama Assemblage. *Geol. Mag.* **160**, 1673–1686 (2023).

67. S. W. F. Grant, Shell structure and distribution of *Cloudina*, a potential index fossil for the terminal Proterozoic. *Am. J. Sci.* **290-A**, 261–294 (1990).

68. X. Wang, A. G. Liu, Z. Chen, C. Wu, Y. Liu, B. Wan, K. Pang, C. Zhou, X. Yuan, S. Xiao, A late-Ediacaran crown-group sponge animal. *Nature* **630**, 905–911 (2024).

69. S. A. F. Darroch, A. T. Cribb, L. A. Buatois, G. J. B. Germs, C. G. Kenchington, E. F. Smith, H. Mocke, G. R. O'Neil, J. D. Schiffbauer, K. M. Maloney, R. A. Racicot, K. A. Turk, B. M. Gibson, J. Almond, B. Koester, T. H. Boag, S. M. Tweedt, M. Laflamme, The trace fossil record of the Nama Group, Namibia: Exploring the terminal Ediacaran roots of the Cambrian explosion. *Earth Sci. Rev.* **212**, 103435 (2021).

70. S. Willman, B. J. Slater, Late Ediacaran organic microfossils from Finland. *Geol. Mag.* **158**, 2231–2244 (2021).

71. R. P. Anderson, N. J. Tosca, E. E. Saupe, J. Wade, D. E. G. Briggs, Early formation and taphonomic significance of kaolinite associated with Burgess Shale fossils. *Geology* **49**, 355–359 (2021).

72. W. Wang, C. Guan, X. Zhao, Y. Fang, C. Zhou, X. Yuan, Taphonomic study of Chuaria fossils from the Ediacaran Lantian biota of South China. *Precambrian Res.* **369**, 106529 (2022).

73. Q. Ye, J. Tong, Z. An, J. Hu, L. Tian, K. Guan, S. Xiao, A systematic description of new macrofossil material from the upper Ediacaran Miaohe Member in South China. *J. Syst. Palaeontol.* **17**, 183–238 (2019).

74. S. Xiao, X. Yuan, M. Steiner, A. H. Knoll, Macroscopic carbonaceous compressions in a terminal Proterozoic shale: A systematic reassessment of the Miaohe biota, South China. *J. Paleontol.* **76**, 347–376 (2002).

75. S. Xiao, Z. Chen, K. Pang, C. Zhou, X. Yuan, The Shibantan Lagerstätte: Insights into the Proterozoic–Phanerozoic transition. *J. Geol. Soc. Lond.* **178**, 2020–2135 (2021).

76. G. Li, F. Wei, W. Wen, X. Wang, X. Lei, R. P. Anderson, Y. Zhao, F. S. Dunn, L. A. Parry, P. Cong, The dawn of the Phanerozoic: A transitional fauna from the late Ediacaran of Southwest China. *Science* **392**, 63–68 (2026).

77. I. Bobrovskiy, J. M. Hope, B. J. Nettersheim, J. K. Volkman, C. Hallmann, J. J. Brocks, Algal origin of sponge sterane biomarkers negates the oldest evidence for animals in the rock record. *Nat. Ecol. Evol.* **5**, 165–168 (2021).

78. G. D. Love, R. E. Summons, The molecular record of Cryogenian sponges–a response to Antcliffe (2013). *Palaeontology* **58**, 1131–1136 (2015).

79. D. A. Gold, S. S. O'Reilly, G. Luo, D. E. G. Briggs, R. E. Summons, Prospects for sterane preservation in sponge fossils from museum collections and the utility of sponge biomarkers for molecular clocks. *Bull. Peabody Mus. Nat. Hist.* **57**, 181–189 (2016).

80. D. A. Gold, J. Grabenstatter, A. de Mendoza, A. Riesgo, I. Ruiz-Trillo, R. E. Summons, Sterol and genomic analyses validate the sponge biomarker hypothesis. *Proc. Natl. Acad. Sci. U.S.A.* **113**, 2684–2689 (2016).

81. E. A. Sperling, R. G. Stockey, The temporal and environmental context of early animal evolution: Considering all the ingredients of an "explosion". *Integr. Comp. Biol.* **58**, 605–622 (2018).

82. J. W. Brown, S. A. Smith, The past sure is tense: On interpreting phylogenetic divergence time estimates. *Syst. Biol.* **67**, 340–353 (2018).

83. N. J. Butterfield, Exceptional fossil preservation and the Cambrian Explosion. *Integr. Comp. Biol.* **43**, 166–177 (2003).

84. L. G. Tarhan, A. v. S. Hood, M. L. Droser, Elevated marine dissolved silica levels explain a wide range of Ediacaran–Cambrian Ediacara-style fossil deposits. *Geobiology* **23**, e70017 (2025).

85. C. R. Woltz, R. P. Anderson, N. J. Tosca, S. M. Porter, The role of clay minerals in the preservation of Precambrian organic-walled microfossils. *Geobiology* **21**, 708–724 (2023).

86. F. Saleh, J. B. Antcliffe, B. Lefebvre, B. Pittet, L. Laibl, F. P. Peris, L. Lustri, P. Gueriau, A. C. Daley, Taphonomic bias in exceptionally preserved biotas. *Earth Planet. Sci. Lett.* **529**, 115873 (2020).

87. L. Parry, J.-B. Caron, *Canadia spinosa* and the early evolution of the annelid nervous system. *Sci. Adv.* **5**, eaax5858 (2019).

88. E. U. Hammarlund, M. P. Smith, J. A. Rasmussen, A. T. Nielsen, D. E. Canfield, D. A. T. Harper, The Sirius Passet Lagerstätte of North Greenland—A geochemical window on early Cambrian low-oxygen environments and ecosystems. *Geobiology* **17**, 12–26 (2019).

89. K. Shimizu, M. Nishi, Y. Sakate, H. Kawanami, T. Bito, J. Arima, L. Leria, M. Maldonado, Silica-associated proteins from hexactinellid sponges support an alternative evolutionary scenario for biomineralization in Porifera. *Nat. Commun.* **15**, 181 (2024).

90. J. L. Payne, C. R. McClain, A. G. Boyer, J. H. Brown, S. Finnegan, M. Kowalewski, R. A. Krause Jr., S. K. Lyons, D. W. McShea, P. M. Novack-Gottshall, F. A. Smith, P. Spaeth, J. A. Stempien, S. C. Wang, The evolutionary consequences of oxygenic photosynthesis: A body size perspective. *Photosynth. Res.* **107**, 37–57 (2010).

91. L. A. Levin, Oxygen minimum zone benthos: Adaptation and community response to hypoxia. *Oceanogr. Mar. Biol.* **41**, 1–45 (2003).

92. G. O. Poinar, *The Evolutionary History of Nematodes: As Revealed in Stone, Amber and Mummies* (Brill, 2011), vol. 9.

93. J. A. Dunlop, R. J. Garwood, Terrestrial invertebrates in the Rhynie chert ecosystem. *Philos. Trans. R. Soc. B Biol. Sci.* **373**, 20160493 (2018).

94. R. P. Anderson, C. R. Woltz, N. J. Tosca, S. M. Porter, D. E. G. Briggs, Fossilisation processes and our reading of animal antiquity. *Trends Ecol. Evol.* **38**, 1060–1071 (2023).

95. T. Brunet, N. King, The origin of animal multicellularity and cell differentiation. *Dev. Cell* **43**, 124–140 (2017).

96. S. D. Evans, C. Tu, A. Rizzo, R. L. Surprenant, P. C. Boan, H. McCandless, N. Marshall, S. Xiao, M. L. Droser, Environmental drivers of the first major animal extinction across the Ediacaran White Sea-Nama transition. *Proc. Natl. Acad. Sci. U.S.A.* **119**, e2207475119 (2022).

97. K. Pehr, G. D. Love, A. Kuznetsov, V. Podkovyrov, C. K. Junium, L. Shumlyanskyy, T. Sokur, A. Bekker, Ediacara biota flourished in oligotrophic and bacterially dominated marine environments across Baltica. *Nat. Commun.* **9**, 1807 (2018).

98. J. E. Johnson, A. Gerpheide, M. P. Lamb, W. W. Fischer, O_2 constraints from Paleoproterozoic detrital pyrite and uraninite. *Geol. Soc. Am. Bull.* **126**, 813–830 (2014).

99. K. E. Tingle, S. M. Porter, M. R. Raven, A. D. Czaja, S. M. Webb, B. Bloeser, Organic preservation of vase-shaped microfossils from the late Tonian Chuar Group, Grand Canyon, Arizona, USA. *Geobiology* **21**, 290–309 (2023).

100. C. Loron, M. Moczydłowska, Tonian (Neoproterozoic) eukaryotic and prokaryotic organic-walled microfossils from the upper Visingsö Group, Sweden. *Palynology* **42**, 220–254 (2018).

101. N. J. Planavsky, O. J. Rouxel, A. Bekker, S. V. Lalonde, K. O. Konhauser, C. T. Reinhard, T. W. Lyons, The evolution of the marine phosphate reservoir. *Nature* **467**, 1088–1090 (2010).

102. W. W. Crockett, J. O. Shaw, C. Simpson, C. P. Kempes, Physical constraints during Snowball Earth drive the evolution of multicellularity. *Proc. R. Soc. B* **291**, 20232767 (2024).

103. E. S. C. Anttila, F. A. Macdonald, D. Szymanowski, B. Schoene, A. Kylander-Clark, C. Danhof, D. S. Jones, Timing and tempo of organic carbon burial in the Monterey Formation of the Santa Barbara Basin and relationships with Miocene climate. *Earth Planet. Sci. Lett.* **620**, 118343 (2023).

104. Z. Yang, PAML 4: Phylogenetic analysis by maximum likelihood. *Mol. Biol. Evol.* **24**, 1586–1591 (2007).

105. S. Q. Le, O. Gascuel, An improved general amino acid replacement matrix. *Mol. Biol. Evol.* **25**, 1307–1320 (2008).

106. L. L. Nelson, J. L. Crowley, E. F. Smith, D. M. Schwartz, E. B. Hodgin, M. D. Schmitz, Cambrian explosion condensed: High-precision geochronology of the lower Wood Canyon Formation, Nevada. *Proc. Natl. Acad. Sci. U.S.A.* **120**, e2301478120 (2023).

107. Y. E. Demidenko, E. Zhegallo, P. Y. Parkhaev, Y. V. Shuvalova, Age of phosphorites from the Khubsugul Basin (Mongolia). *Doklady Earth Sci.* **389**, 317–321 (2003).
